# Identification and Characterization of a Novel Emaravirus From Grapevine Showing Chlorotic Mottling Symptoms

**DOI:** 10.3389/fmicb.2021.694601

**Published:** 2021-06-07

**Authors:** Xudong Fan, Chen Li, Zunping Zhang, Fang Ren, Guojun Hu, Hailin Shen, Baodong Zhang, Yafeng Dong

**Affiliations:** ^1^National Center for Eliminating Viruses from Deciduous Fruit Trees, Research Institute of Pomology, Chinese Academy of Agriculture Sciences, Xingcheng, China; ^2^Pomology Research Institute, Jilin Academy of Agricultural Sciences, Gongzhuling, China

**Keywords:** grapevine, high-throughput sequencing, chlorotic mottling, *Emaravirus*, genome, virus

## Abstract

A novel negative-sense, single-stranded (ss) RNA virus was identified in a “Shennong Jinhuanghou” (SJ) grapevine showing severe chlorotic mottling symptoms by integrating high-throughput sequencing (HTS) and conventional Sanger sequencing of reverse transcription polymerase chain reaction (RT-PCR) products. The virus was provisionally named as “grapevine emaravirus A” (GEVA). GEVA had a genome comprising five genomic RNA segments, each containing a single open reading frame on the viral complementary strand and two untranslated regions with complementary 13- nt stretches at the 5′ and 3′ terminal ends. RNA1 (7,090 nt), RNA2 (2,097 nt), RNA3 (1,615 nt), and RNA4 (1,640 nt) encoded putative proteins P1–P4 that, based on their conserved motifs, were identified as the RNA-dependent RNA polymerase, glycoprotein, nucleocapsid protein, and movement protein, respectively. However, the functional role of protein P5 encoded by RNA5 (1,308 nt) could not be determined. Phylogenetic trees constructed based on amino acids of P1 to P4, allocated GEVA in clade I, together with other species-related emaraviruses. These data support the proposal that GEVA is a representative member of a novel species in the genus *Emaravirus* of the family *Fimoviridae*. Moreover, when GEVA was graft-transmitted to SJ and “Beta” grapevines, all grafted plants showed the same symptoms, similar to those observed in the source of the inoculum. This is the first report to our knowledge of an emaravirus infecting grapevine and its possible association with chlorotic mottling symptoms.

## Introduction

*Emaravirus* is the sole genus in the family *Fimoviridae* and encompasses plant viruses with multiple, negative-sense, single-stranded (ss) RNA genomes (Muhlbach and Mielke-Ehret, 2012; Elbeaino et al., 2018). Viruses belonging to twenty-one approved species have been assigned to the genus *Emaravirus*: Actinidia chlorotic ringspot-associated virus (AcCRaV) (Zheng et al., 2017b), blackberry leaf mottle-associated virus (BLMaV) (Hassan et al., 2017), European mountain ash ringspot-associated virus (EMARaV; representing the type species of the genus *Emaravirus*) (Mielke-Ehret and Muehlbach, 2007; von Bargen et al., 2019), fig mosaic virus (FMV) (Elbeaino et al., 2009a,b; Ishikawa et al., 2012), High Plains wheat mosaic virus (HPWMoV) (Tatineni et al., 2014), pigeonpea sterility mosaic virus 1 (PPSMV-1) (Elbeaino et al., 2014), PPSMV-2 (Elbeaino et al., 2015), Pistacia emaravirus B (PiVB) (Buzkan et al., 2019), raspberry leaf blotch virus (RLBV) (McGavin et al., 2012), redbud yellow ringspot-associated virus (RYRSaV) (Di Bello et al., 2016), rose rosette virus (RRV) (Laney et al., 2011), Actinidia virus-2 (AcV-2) (Wang Y.X. et al., 2020), aspen pistacia mosaic-associated virus (AsMaV) (von Bargen et al., 2020), lilac chlorotic ringspot-associated virus (LiCRaV) (Wang Y.Q. et al., 2020), blue palo verde broom virus (PVBV) (Ilyas et al., 2018), pear chlorotic leaf spot-associated virus (PCLSaV) (Liu et al., 2020), ti ringspot-associated virus (TiRSaV) (Olmedo-Velarde et al., 2019), Camellia japonica-associated emaravirus 1 (CjaEV1), CjaEV2 (Peracchio et al., 2020; Zhang et al., 2020), jujube yellow mottle-associated virus (JYMaV) (Yang et al., 2019), and Perilla mosaic virus (PerMV) (Kubota et al., 2020). Other tentative emaraviruses have been reported recently: maple mottle-associated virus (MaMaV) (Rumbou et al., 2021), common oak ringspot-associated emaravirus (CORaV) (Rehanek et al., 2021), and Chrysanthemum mosaic-associated virus (ChMaV) (Kubota et al., 2021). Although emaraviruses are reported to be associated with viral disease in some fruits (e.g., *Actinidia* species, blackberry, fig, raspberry, jujube, and pear), they have not been reported in grapevines.

Emaraviruses consist of 4–8 RNA segments in their genomes. Each segment contains a single open reading frame (ORF) on the strand of complementary RNA. The RNA1, RNA2, RNA3, and RNA4 segments of emaraviruses encode an RNA-dependent RNA polymerase (RdRp), a glycoprotein (GP) precursor, a nucleocapsid protein (NP), and a non-structural movement protein (MP), respectively, as core elements (Elbeaino et al., 2018). The proteins encoded by other RNAs (RNA5–RNA8) show low sequence similarity with other functionally identified proteins, and their roles remain to be explored further. The P7 and P8 proteins of HPWMoV have been suggested to utilize distinct mechanisms for overcoming RNA silencing of the host, allowing establishment of a systemic infection (Gupta et al., 2018, 2019).

China is one of the world’s leading grape-production areas (estimated recently to cover a total area of 725,100 hectares). Grapevine virus diseases are major threats to grape production, with 21 species of grapevine viruses reported in China (Fan et al., 2020; Ren et al., 2021). Chlorotic mottling symptoms caused by viruses on grapevines are common, and have become a major problem in China. Symptom investigation and virus identification in many grapevine samples revealed that grapevine berry inner necrosis virus (GINV), grapevine Pinot gris virus (GPGV), and grapevine fabavirus (GFabV) are associated with mottling and/or deformation of grapevine leaves in some cultivars and rootstocks in China (Fan et al., 2017a,b, 2018). However, further investigation is needed to fully identify the viruses associated with symptoms of grapevine mottling.

During a field investigation in 2016, the obvious symptoms of chlorotic mottling on the leaves of a “Shennong Jinhuanghou” (SJ) grapevine cultivar were observed. A preliminary investigation conducted using reverse-transcription polymerase chain reaction (RT-PCR) assay showed that the SJ grapevine did not harbor common viruses identified previously in China. To identify possible virus infections in the diseased-grapevine sample, small RNA sequencing (sRNA-seq) and RNA sequencing (RNA-seq) were used to identify viruses in diseased grapevine samples in 2018.

## Materials and Methods

### Plant Material for High-Throughput Sequencing

**FIGURE 1:**
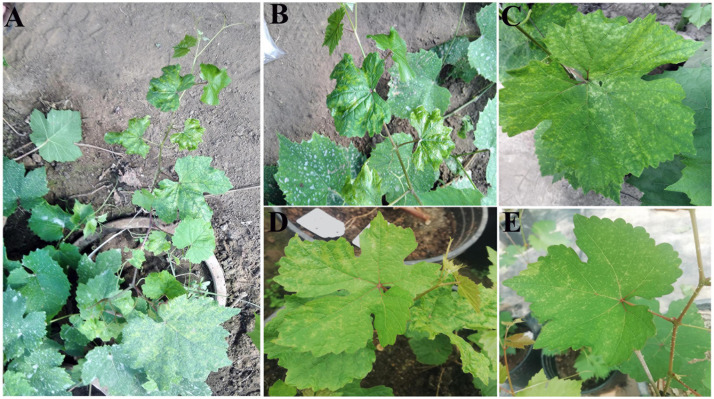
Symptoms on the leaves of a “Shennong Jinhuanghou” (SJ) grapevine infected with grapevine emaravirus A (GEVA). **(A)** GEVA-infected SJ plant; **(B)** chlorotic mottling and shrinking symptoms on GEVA-infected leaves; **(C)** chlorotic mottling symptoms; **(D,E)** chlorotic mottling on leaves of cut seedlings propagated from the GEVA-infected plant.

An SJ grapevine (*Vitis vinifera* L.), showing systemic symptoms of chlorotic mottling (Figures 1A–C) during observation from 2016 to 2018 was preserved in a sample-preservation nursery of the National Center for Eliminating Viruses in Xingcheng City (Liaoning, China). Cut seedlings propagated from the infected grapevine also showed symptoms of chlorotic mottling (Figures 1D,E). In spring 2018, diseased leaves were collected and were frozen rapidly in liquid nitrogen before preserving in carbon dioxide ice-blocks and shipping to Biomarker Biology Technology (Beijing, China), which took 2–3 days.

### sRNA-seq and RNA-seq

Leaf samples were used to extract total RNAs and generate a complementary (c)DNA library of sRNAs. sRNA-seq was carried out using an Illumina HiSeq^TM^ 2000 system (SinoGenoMax, Beijing, China), as reported previously (Fan et al., 2016). “Clean” data were obtained by removing sequences <18 nucleotides (nt) or >30 nt, low-quality tags, poly-A tags, and N tags from raw reads. Sequences of potential viruses were identified by analyses of clean data using VirusDetect^1^ (Zheng et al., 2017a). After preliminary identification of a novel emaravirus by sRNA-seq, the same sample was also used to identify other potential sequences of the newly identified virus by RNA-seq, and also to obtain longer assembled sequences. For RNA-seq, the Epicenter Ribo-Zero rRNA Removal Kit (Epicenter, Madison, WI, United States) was used to remove ribosomal RNA from extracts of total RNA. The ribosomal RNA-depleted RNA sample was then used to construct a cDNA library using a TruSeq RNA Sample Prep Kit (Illumina, San Diego, CA, United States), which was sequenced on an Illumina HiSeq 4000 platform with a paired-end 150-bp setup (Biomarker Biology Technology). Reads mapping to the grapevine genome (PN40024 assembly 12X) were filtered out by hierarchical indexing using *hisat* software (Kim et al., 2015). Unmapped reads were used for *de novo* assembly and Blast analysis embedded in VirusDetect.

### Determination of the GEVA Genome

Overlapping fragments of viral RNAs were amplified by RT-PCR with specific primers (Supplementary Table 1). These primers were designed on the basis of contig sequences and the primers 5H/3C targeting the 13-nt stretches conserved at the 3′ and 5′ termini of genomic RNAs of emaraviruses (Zheng et al., 2017b). Adjacent amplicons overlapped by >100 bp. PCR fragments were recovered and purified, then cloned into the Zero Background pTOPO-Blunt vector (Aidlab, Beijing, China). At least three positive clones of each PCR product were sequenced at Shanghai Sangon Biological Engineering and Technology (Shanghai, China). The sequences obtained were assembled into contiguous sequences by overlapping common regions (in general, ∼100 bp) of the amplicons. The 3′ end of GEVA RNAs was poly(A) tailed using a poly(A) polymerase (catalog number, 2181; TaKaRa Biotechnology, Shiga, Japan). The 5′ and 3′ untranslated regions (UTRs) were determined using the rapid amplification of cDNA ends (RACE) strategy employing a SMARTer^®^ RACE 5′/3′ Kit (catalog number, 6106: TaKaRa Biotechnology) according to the manufacturer’s instructions. Primers 5H/3C usually allow full-length amplification of emaravirus RNAs (expect for RNA1) by RT-PCR (Di Bello et al., 2015). To ascertain if GEVA had other RNA segments, the RNA segments of GEVA were further amplified using 5H/3C primers from the cDNA products generated by RT using 3C primer and 3′-CDS primer provided in the RACE Kit.

### Sequence Analyses

Open reading frame Finder at the National Center for Biotechnology Information (NCBI) was used to search for potential ORFs in the genomic and anti-genomic RNAs of the virus. Nucleotide (nt) and amino acid (aa) sequences were compared with those of other emaraviruses using ClustalW2^2^. Phylogenetic analyses of the coding regions of RNAs 1–4 were performed using Molecular Evolutionary Genetics Analysis (MEGA) 7.0^3^. Protein analyses for prediction of the transmembrane domains of proteins were conducted using TMHMM^4^ (Krogh et al., 2001). Prediction of *N*- glycosylation sites and signaling peptides was conducted using NetNGlyc 1.0^5^. Prediction of cleavage sites was conducted using SignalP 3.0^6^ (Bendtsen et al., 2004).

### Graft-Transmission Assays

Graft transmissibility was assessed by grafting GEVA-infected grapevine buds onto SJ seedlings and “Beta” grapevine seedlings (2 years of age) in four replicates in July 2020. These seedlings had tested negative for GEVA and other major viruses reported in China: grapevine leafroll-associated virus-1 (GLRaV-1), GLRaV-2, GLRaV-3, GLRaV-4, GLRaV-7, GLRaV-13, grapevine rupestris stem pitting-associated virus (GRSPaV), grapevine fleck virus (GFkV), grapevine fanleaf virus (GFLV), grapevine virus A (GVA), grapevine virus B (GVB), grapevine virus E (GVE), grapevine Pinot gris virus (GPGV), grapevine berry inner necrosis virus (GINV), grapevine fabavirus (GFabV), grapevine rupestris vein feathering virus (GRVFV), grapevine geminivirus A (GGVA), grapevine Syrah virus-1 (GSyV-1), and grapevine red globe virus (GRGV). Inoculated seedlings were maintained in a greenhouse under natural conditions for symptom development. Grafted grapevines were observed continuously for symptoms after inoculation. After 2 months, total RNAs were extracted from the leaves of the grafted SJ grapevine and Beta grapevine using the method described by MacKenzie et al. (1997) with light modifications (Fan et al., 2017b). Two primer pairs, P3-1a/1b (5′-GTTAGCTGGAGCCATGGGAA-3′ and 5′-TAGGCGTGTCTGGAAAAGCT-3′) and P4-1a/1b (5′-AAGGGCAACATCAGAGTCAGG-3′ and 5′-TGATGAGGGCTGCTAATGGA-3′), were used to amplify 316- and 236-bp fragments of GEVA RNA3 and RNA4, respectively, and were used in the RT-PCR detection of GEVA in these samples.

### Virus Detection in the Field

To survey GEVA prevalence, 473 grapevine samples of 71 cultivars were collected from 21 provinces in China during 2018–2020. These samples were tested for GEVA by RT-PCR using the primers P3-1a/1b and P4-1a/1b. Amplification products from positive samples were cloned and sequenced as described above.

## Results

### Virus Identification by High-Throughput Sequencing

Sequencing of a cDNA library of sRNAs from symptomatic leaves of the SJ grapevine (Figures 1A,B) resulted in 14,348,642 clean reads. The clean data were used for virus identification using VirusDetect. BLASTN and BLASTX results revealed that seven contigs and two contigs were homologous to grapevine yellow speckle viroid 1 (GYSVd1) and hop stunt viroid (HSVd), respectively, and 12 contigs of length 59–391 nt were homologous to P1-P4 proteins of several emaraviruses and P6 of PPSMV-2 (Supplementary Tables 2, 3). These results indicated the presence of a candidate emaravirus in the SJ grapevine sample, which we tentatively named as grapevine emaravirus A (GEVA). To obtain more sequences of GEVA, we performed RNA-seq analyses for the same sample. High-quality clean data comprising 60,208,348 reads were generated by RNA-seq, and BLASTX analyses identified 10 contigs of length 271–2,069 nt homologous to P1–P4 and P6 proteins of emaraviruses (Supplementary Tables 2, 3); their BLASTX coverage on the aa sequences of P1, P2, P3, P4, and P6 was 99.5, 98.9, 100, 93.1, and 44.1%, respectively. Together, we identified partial sequences of five potential RNAs of GEVA through sRNA-seq and RNA-seq analyses.

### Determination and Analyses of the GEVA Genome

**FIGURE 2:**
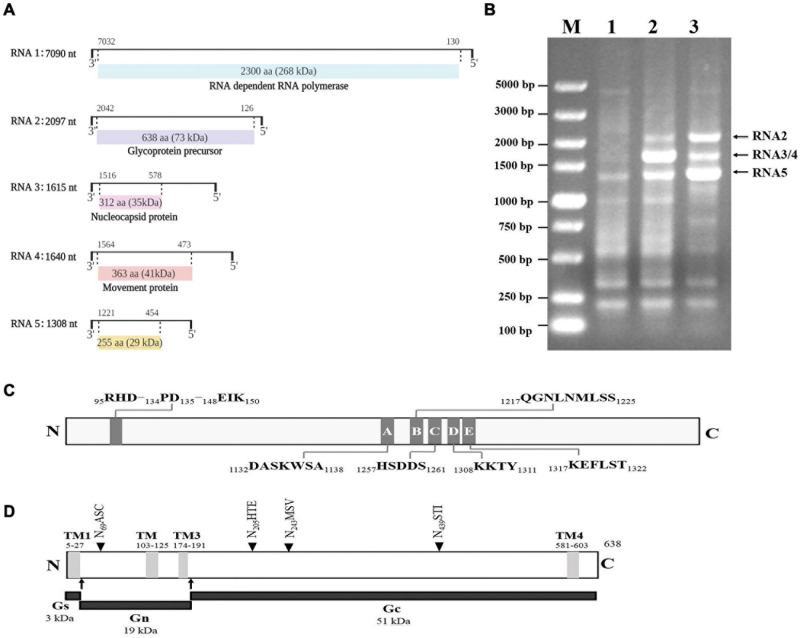
Genomic organization of grapevine emaravirus A (GEVA) (schematic) **(A)**. The nucleotide (nt) length of each RNA is represented as a black line. Black boxes at the end of each line indicate the 13 nt conserved at RNA termini. The expression product of each RNA is shown as a box, with the amino acid (aa) length, estimated molecular mass (kDa) and putative protein indicated. **(B)** Reverse transcription polymerase chain reaction (RT-PCR) of full-length RNA genomic segments of GEVA. Lane 1, asymptomatic leaves of a “Shennong Jinhuanghou” (SJ) grapevine not infected by GEVA; lane 2, symptomatic leaves of a SJ grapevine infected by GEVA, with RT using the 3C primer; lane 3, symptomatic leaves of a SJ grapevine infected by GEVA, with RT using the 3′-CDS primer provided with the RACE kit (TaKaRa Biotechnology, Shiga, Japan); M, DNA molecular mass marker with sizes (bp) indicated on the left. **(C)** Conserved endonuclease domain and motifs A–E in the RdRp of GEVA. **(D)** Conserved transmembrane domains (TMs), three glycoproteins, and four putative N-glycosylation sites (NASC, NHTE, NMSV, NSTI) in the GP of GEVA.

RNA1, RNA2, RNA3, RNA4, and RNA5 had lengths of 7,090, 2,097, 1,615, 1,640, and 1,308 nt, respectively (Figure 2A). The first 13 nt at the 5′ termini (AGUAGUGUUCUCC) and at the 3′ termini (GGAGUUCACUACU) of the RNA segments were almost complementary to each other and conserved in the five viral genomic RNA components. We amplified three obvious bands using primers 5H/3C from the two types of positive cDNAs (Figure 2B), and sequencing confirmed that these had sequences specific to GEVA representing the segments of RNA2, RNA3/RNA4, and RNA5. All attempts to amplify additional RNA segments for GEVA were unsuccessful, suggesting that GEVA genome consists of five RNA segments.

RNA1 was 7,090 nt in length and contained an ORF (ORF1; nt positions 7,032 to 130) encoding a putative protein (P1) of 2,300 aa with a predicted molecular mass of 268 kDa. The sequence identity between GEVA P1 and RdRps of other emaraviruses ranged from 27.8 to 49.8% (Supplementary Table 4). P1 of GEVA contained conserved motifs (A to E) (Figure 2C and Supplementary Figure 1) within bunyavirus replicases (Reguera et al., 2010; Elbeaino et al., 2013), from aa 1,132 to aa 1,322. Motifs A (DASKWSA_1132__–__1138_) and C (HSDDS_1257__–__1261_) are part of the palm domain of the replicase, and are involved in the binding of divalent metal cations (Bruenn, 2003). Motif B (QGNLNMLSS_1217__–__1225_) is thought to be involved in RNA binding with the Gly residue, which allows for mobility in the peptide backbone; motif D (KKTY_1308__–__1311_) has a strictly conserved Lys residue, and is proximal to the Asp of motif A (Lukashevich et al., 1997). Motif E (KEFLST_1317__–__1322_) is likely to be involved in “cap-snatching” in bunyaviruses as well as in possible endonuclease activity (Laney et al., 2011). P1 also contained an endonuclease domain in the N-terminus (RHD_95__–__97_–PD_134__–__135_–EIK_148__–__150_), which is deemed to be involved in cap-snatching of viral mRNAs during genome expression (Juan et al., 2010). Such results from other research groups support the prediction that GEVA P1 is a homolog of emaravirus RdRps.

RNA2 was 2,097 bp in length and contained an ORF (nt positions 2,042 to 126) encoding P2, a putative GP of 638 aa with a predicted molecular mass of 73 kDa. GEVA P2 shared identity of 19.0–43.1% at the aa level with GPs of the other emaraviruses. *In silico* analyses predicted four transmembrane helices (at aa positions 5–27, 103–105, 174–191, and 581–603) and four N-glycosylation sites (N_69_ASC, N_205_HTE, N_243_MSV, and N_439_STI) (Figure 2D and Supplementary Figure 2). Two cleavage sites (K_28_L/VNV and K_192_A/DDN) that would process the GP precursor into the C-terminal GP (Gc, 51 kDa) and two N-terminal GPs, a larger Gn (19 kDa) and a smaller Gs (3 kDa) (Mielke-Ehret and Mühlbach, 2012), were also predicted.

RNA3 was of length 1,615 nt with an ORF (nt positions 1,516 to 578) encoding P3, a putative NP of 313 aa with a predicted molecular mass of 35 kDa. It shared aa-sequence identity of 18.5 to 43.5% with NPs of the other emaraviruses. Sequence alignment of P3 identified three conserved aa blocks (XXVSFNKACA_136__–__145_, NRLA_184__–__187_, and GXEF_205__–__208_) similar to the NP motifs reported in other emaraviruses (Elbeaino et al., 2009b) and which have been predicted to be involved in RNA binding (Tatineni et al., 2014).

RNA4 was 1,640 nt in length and contained a single ORF (nt positions 1,564 to 473) encoding P4, a putative protein of 363 aa with a predicted molecular mass of 41 kDa. It shared aa-sequence identity of 11.2–34.7% with the NPs of the other emaraviruses. We identified a signal peptide with a cleavage site (VKA23DD) using SignalP (Emanuelsson et al., 2007). SMART and CDD analyses showed that the central part of GEVA-P4 contains structural elements similar to the consensus secondary structure of the 30K superfamily of plant virus MPs (pfam16505; interval: 26–360; E-value: 6.46e-99).

RNA5 was of length 1,308 nt and had one ORF (nt positions 1,221 to 454) encoding the P5 protein consisting of 255 aa with a molecular mass of 29 kDa. A BLASTp search against the GenBank database of NCBI indicated that GEVA P5 shared a limited aa-sequence identity of 25.8% with PiVB P6, but did not display significant sequence identity with proteins encoded by other emaraviruses or the proteins available in GenBank. Moreover, we did not identify a conserved domain in P5 through CDD analysis. Thus, we could not ascertain the function of this viral protein.

### Distribution of GEVA-Derived sRNA and RNA Reads Along the GEVA Genome

**FIGURE 3:**
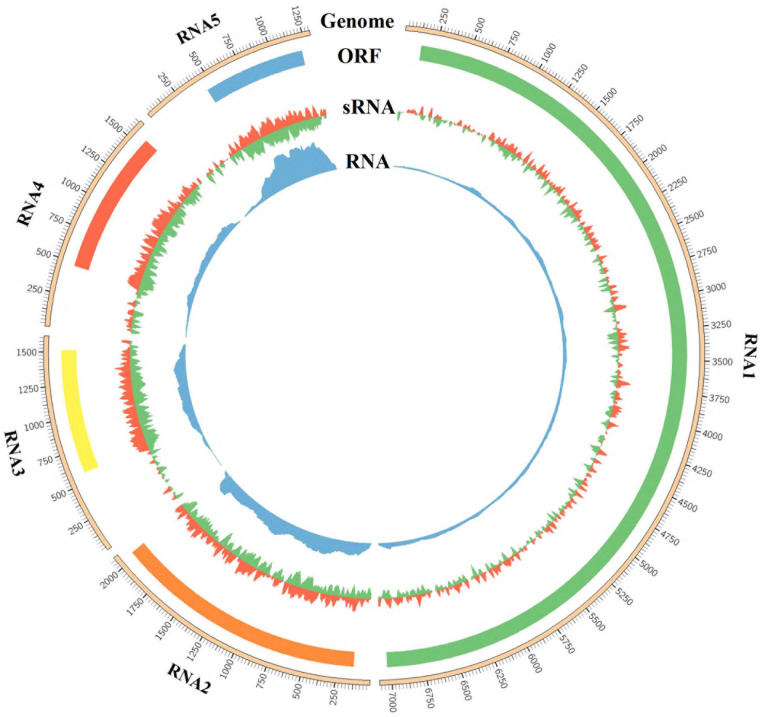
Profile distribution of reads from sRNA-seq and RNA-seq libraries along RNA1 to RNA5 of grapevine emaravirus A (GEVA). Outer ring represents the genome of RNA1, RNA2, RNA3, RNA4, and RNA5 segments of GEVA; the open reading frame (ORF) in each RNA segment is shown below the genome graphic and above the depth of sRNA reads and RNA reads.

Mapping results showed that 23,829 (0.17%), 61,088 (0.43%), 52,943 (0.37%), 86,093 (0.60%), and 79,211 (0.55%) of 14,348,642 clean reads were derived from RNA1, RNA2, RNA3, RNA4, and RNA5, respectively (Supplementary Table 5). The sRNA reads of the clean data could almost exactly covered each segment of RNA1–RNA5 (Figure 3). Most RNA reads were 18–22 nt in length, with a prominent peak corresponding to a size of 21 nt observed for all RNAs (Supplementary Figure 3). There was no significant difference in the proportion of 22-nt or 20-nt reads, but both were significantly lower than the number of reads measuring 21 nt. The “hotspots” of GEVA sRNAs were mapped within ORFs, with far fewer hotspot sRNAs located in the UTRs for RNAs 3, 4, and 5. The 5′-terminal nt of sRNAs showed an inconspicuous bias regardless of polarity or size; U was dominant in all RNAs, but C instead of U in the anti-genome of RNA4 was dominant for 21-nt sRNAs (Supplementary Figure 4). RNA-seq revealed that 224,226 (0.37%) of 60,846,756 clean reads mapped along the GEVA genome; reads were abundant in RNA5 but were far fewer in RNA4 as in comparison, that is similar to results from a study by Liu et al. (2020).

### Phylogenetic Relationships of GEVA With Other Emaraviruses

**FIGURE 4:**
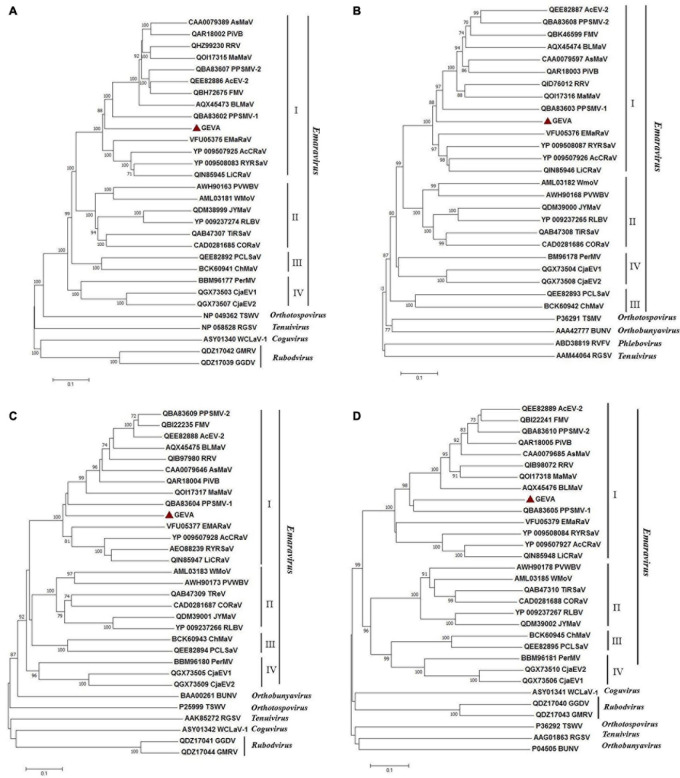
Unrooted neighbor-joining (NJ) phylogenetic trees generated from the deduced amino-acid sequences of the RNA-dependent RNA polymerase (P1) **(A)**, glycoprotein precursor (P2) **(B)**, nucleocapsid protein (P3) **(C)**, and movement protein (P4) **(D)** of emaraviruses. Orthologs from representative members of the genera *Orthotospovirus*, *Orthobunyavirus*, *Coguvirus*, *Tenuivirus*, *Rubodvirus*, and *Phlebovirus* are included in these trees. The bar represents the number of amino-acid replacements per site. GenBank accession numbers of proteins used for phylogenetic analyses are reported. Grapevine emaravirus A (GEVA) is highlighted by a red triangle. FMV, fig mosaic virus; PPSMV-1, pigeonpea sterility mosaic virus 1; PPSMV-2, pigeonpea sterility mosaic virus 2; PiVB, Pistacia virus B; RRV, rose rosette virus; BLMaV, blackberry leaf mottle-associated virus; EMARaV, European mountain ash ringspot-associated virus; AcCRaV, Actinidia chlorotic ringspot-associated virus; RYRSaV, redbud yellow ringspot-associated virus; RLBV, raspberry leaf blotch virus; PVWBV, palo verde witches broom virus; WMoV, wheat mosaic virus; AcEV-2, Actinidia emaravirus 2; JYMaV, jujube yellow mottle associated virus; TiRSaV, ti ringspot-associated virus; AsMaV, aspen mosaic-associated virus; LiCRaV, lilac chlorotic ringspot-associated virus; PerMV, Perilla mosaic virus; PCLSaV, pear chlorotic leaf spot-associated virus. TSWV, tomato spotted wilt virus; BUNV, bunyamwera virus; WCLaV-1, watermelon crinkle leaf-associated virus 1; RGSV, rice grassy stunt virus; GMRV: Muscat rose virus; GGDV, grapevine Garan dmak virus; RVFV, Rift Valley fever virus.

Four phylogenetic trees were reconstructed in order to establish the relationship between GEVA and other emaraviruses (Figure 4). Amino acid sequences of putative RdRp (P1), GP (P2), CP (P3), and MP (P4) proteins from GEVA, all known emaraviruses, and several representative members of the genera *Phlebovirus*, *Tenuivirus*, *Coguvirus*, and *Rubodvirus* in the family *Phenuiviridae*, the genus *Orthotospovirus* in the family *Tospoviridae*, and the genus *Orthobunyavirus* in the family *Bunyaviridae* were used in the tree reconstruction. Regardless of the proteins under consideration, GEVA clustered together with emaraviruses with high bootstrap values, and was clearly separated from other viruses in several genera in the order *Bunyavirales*, thereby confirming a close phylogenetic relationship of GEVA with members of the genus *Emaravirus* (Figure 4). In P1-, P2-, P3-, and P4-based trees, GEVA isolates clustered consistently within clade I, as established previously (Liu et al., 2020), but formed a separate clade from other emaraviruses in the group.

### Graft-Transmission of GEVA

**FIGURE 5:**
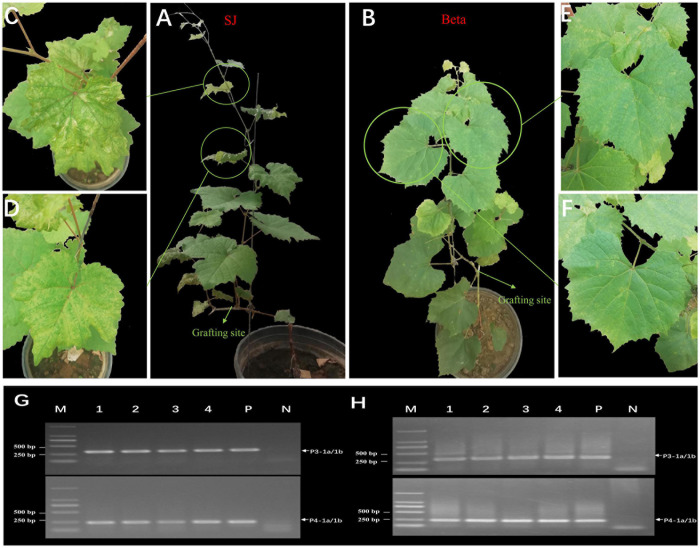
Mechanical transmission of grapevine emaravirus A (GEVA) to grapevines. **(A)** Grafted “Shennong Jinhuanghou” (SJ) grapevine; **(B)** grafted “Beta” grapevine; Panels **(C,D)** partially enlarged image of panel **(A)**; Panels **(E,F)** partially enlarged image of panel **(B)**. **(G,H)** RT-PCR detection of GEVA in grafted grapevines using two sets of primers. Lane 1–4, grafted grapevine samples; P, positive control; N, negative control; M, DNA molecular-weight marker with sizes (bp) indicated on the left.

All grafted SJ and Beta grapevines showed obvious symptoms of chlorotic mottling on the leaves of the first bud below the grafting site 2 months after grafting (Figures 5A–F). We confirmed the presence of GEVA in grafted plants by RT-PCR using two sets of primers that amplified the conserved regions of the GEVA RNA3 and RNA4 genomes (Figures 5G,H). By contrast, non-grafted plants tested negative for GEVA and did not elicit symptoms. These data suggested that GEVA could be transmitted by grafting, and may be associated with the chlorotic mottling symptoms observed in SJ and Beta grapevines.

### Detection and Prevalence of GEVA in the Field

Among all samples tested, three SJ grapevine samples from Jilin Province tested positive for GEVA. The other 470 samples tested negative for GEVA. Sequencing results revealed nt-sequence identity of 99.4–100% between the amplicon sequences of GEVA from the three positive samples.

## Discussion

High-throughput sequencing (HTS) has become very popular in recent years for identification of potentially novel viruses associated with fruit disease (Maliogka et al., 2018). Several viruses infecting grapevines have been identified using HTS in the last decade. Some of them, such as GPGV (Saldarelli et al., 2015) and GRBV (Sudarshana et al., 2015), can cause serious disease in grapevines. Here, we demonstrated by sRNA-seq and RNA-seq, for the first time to our knowledge, infection by a novel emaravirus, GEVA, in a grapevine sample showing chlorotic mottling symptoms. Our results expand knowledge of the host range and disease caused by viruses in the genus *Emaravirus*.

Five genomic components of GEVA from a SJ grapevine sample were determined by integrating data from sRNA-seq, RNA-seq, and conventional Sanger-seq. Each genomic RNA of GEVA possessed a single ORF on its complementary strand and 13-nt stretches with complementary sequences at its 5′ and 3′ termini, similar to those of other emaraviruses (Zheng et al., 2017b). The P1–P4 proteins encoded by GEVA were presumed to be the viral RdRp, GP, CP, and MP due to the presence of similar domains and motifs in homologous proteins from other emaraviruses, however, there was low overall sequence identity of P1–P4 from GEVA with these proteins from other emaraviruses. Furthermore, the phylogenetic tree of RdRp, GP, CP, and MP showed that GEVA was positioned in subgroup I of *Emaravirus*, as reported by Liu et al. (2020), but which was subdivided into subgroups I and II in studies by other researchers (Kubota et al., 2020; Wang Y.X. et al., 2020). These characteristics strongly support the notion of GEVA being a novel member of the genus *Emaravirus*. To ascertain if GEVA had more than five types of RNA, we used the primers 5H/3C to amplify the full-length of emaravirus RNA according to the method described by Di Bello et al. (2015). As reported previously, we were able to amplify four of five viral RNA segments, and did not find additional RNA segments of GEVA, confirming that the GEVA genome has five RNA segments.

The depth of sRNA and RNAs from RNA1 was lower than that for other RNAs, and a greater depth of sRNA reads and RNA reads was present for RNA5, similar to that noted previously (Zheng et al., 2017b; Liu et al., 2020). The distribution profile of hotspots of GEVA sRNAs (which were high in ORFs and low in UTRs for RNA3–RNA5) was the same as that reported by Zheng et al. (2017b) (Liu et al., 2020) and was suggested to be a common feature of the genus *Emaravirus*. Analyses of the sRNAs derived from the GEVA genome revealed that most were 21 nt in length, suggesting that DICER-like (DCL) enzyme-4 had a major role in siRNA production (Bouche et al., 2006; Garcia-Ruiz et al., 2010). Bouche et al. (2006) also showed that viral siRNAs are produced mainly by DCL4, and that DCL2 can substitute for DCL4 if the activity of the latter is reduced/inhibited by viruses. This substitution effect may have been less pronounced in the GEVA-infected sample because the peak of 22-nt virus-derived sRNA was not large. Similar to AcCRaV (Zheng et al., 2017b), U was the most abundant nucleotide at the 5′-end of GEVA-derived sRNAs. Nevertheless, while U was abundant at the 5′-end of 21-nt sRNAs for RNA1–RNA3 and RNA5 of GEVA, C was abundant in 21-nt sRNA from RNA4. These data suggest that sRNAs from RNA4 may be loaded by different AGO-containing complexes depending on the polarity (Mi et al., 2008).

Chlorotic mottling of leaves is a common virus-caused symptom in grapevines in China. Fan and coworkers suggested that GPGV, GINV, and GFabV may cause similar symptoms (Fan et al., 2020). However, we found only GEVA and two viroids in the SJ grapevine having chlorotic-mottling symptoms according to HTS, and not the viruses mentioned above. Furthermore, we surveyed GEVA pathogenicity though grafting, and observed similar symptoms in grafted SJ and Beta grapevines. These findings suggest that GEVA may be associated with the disease in SJ grapevine. Nevertheless, GEVA pathogenicity alone is difficult to determine because GEVA was co-infected with viroids in all the samples tested. The field survey showed that GEVA was present in only a few SJ grapevines, and had not spread widely. However, the potential harm of GEVA to grapevines should be a cause for concern considering its grafting transmissibility and potential vector transmissibility.

## Data Availability Statement

The data presented in the study are deposited in the NCBI repository (https://www.ncbi.nlm.nih.gov/), accession number were MW888853–MW888857.

## Author Contributions

XF and YD conceived and designed the experiments. XF, ZZ, and HS collected the samples. XF, CL, FR, GH, BZ, and YD conducted the experiments and analyzed data. XF, CL, and YD discussed the results and drafted and revised the manuscript. All authors approved the final draft of the manuscript.

## Conflict of Interest

The authors declare that the research was conducted in the absence of any commercial or financial relationships that could be construed as a potential conflict of interest.

## Footnotes

1 http://virusdetect.feilab.net/cgi-bin/virusdetect/index.cgi/

2 www.ebi.ac.uk/Tools/msa/clustalw2/

3 www.megasoftware.net/

4 www.cbs.dtu.dk/services/TMHMM/

5 www.cbs.dtu.dk/services/NetNGlyc/

6 www.cbs.dtu.dk/services/SignalP-3.0/

## Supplementary Material

Supplementary Figure 1 Conserved motifs A, B, C, D, and E in P1 of grapevine emaravirus A (GEVA) and other emaraviruses.

Supplementary Figure 2 Conserved motifs in P3 of grapevine emaravirus (GEVA) and other emaraviruses.

Supplementary Figure 3 Size distribution of 18- to 30-nt sRNAs derived from grapevine emaravirus A (GEVA) positive- and anti-genomic RNA strands.

Supplementary Figure 4 Relative frequency of 5′-terminal nucleotides of 18–30 nt grapevine emaravirus A (GEVA) sRNAs.
