# Supplementary figures and images for: Identification and Characterization of a Novel Emaravirus From Grapevine Showing Chlorotic Mottling Symptoms
Source: Front Microbiol. 2021 Jun 7;12:694601. doi: 10.3389/fmicb.2021.694601 (PMC8215277; doi:10.3389/fmicb.2021.694601)

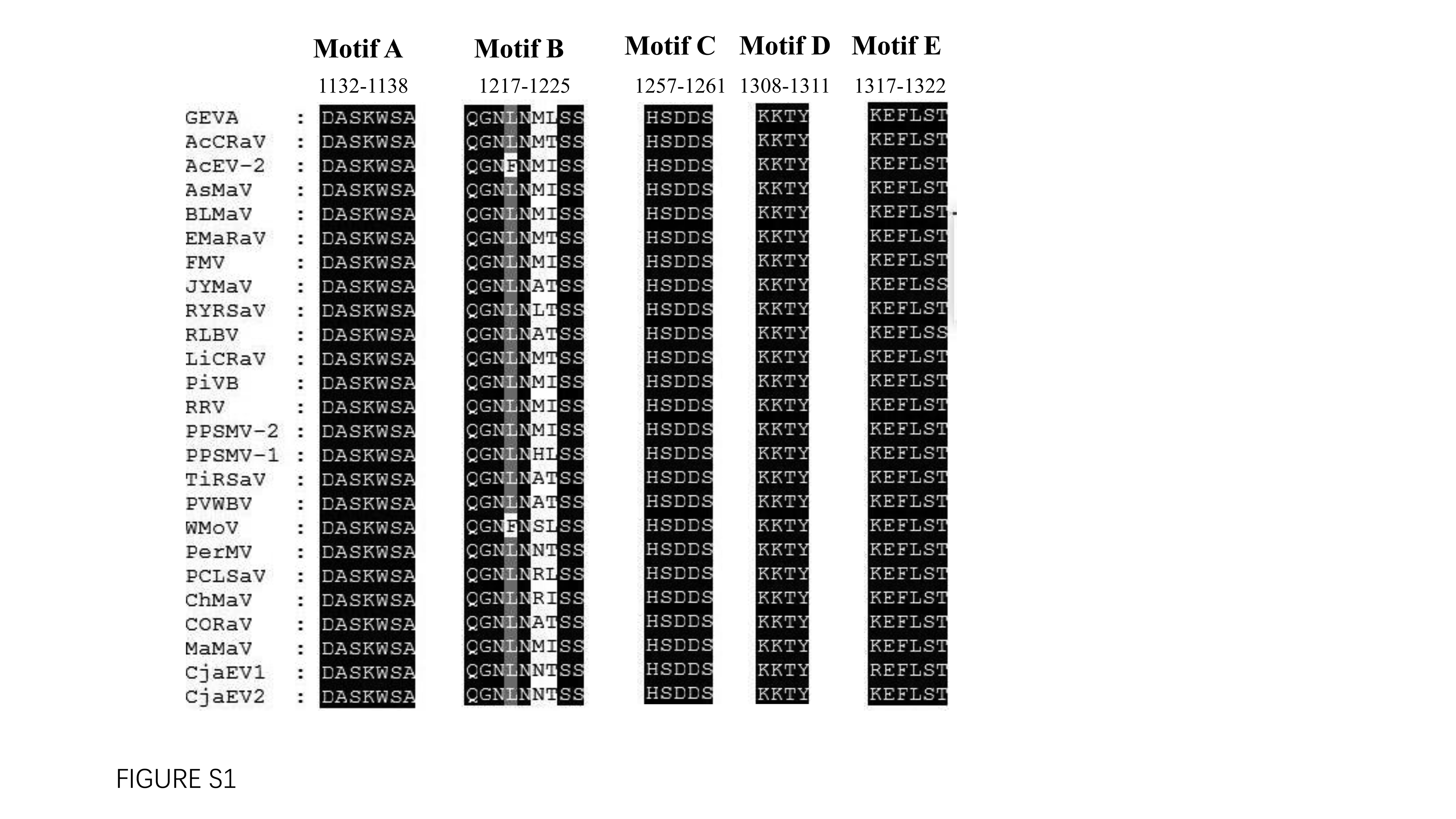

Supplement: Supplementary Figure 1 — Conserved motifs A, B, C, D, and E in P1 of grapevine emaravirus A (GEVA) and other emaraviruses. [file Image_1.TIF]

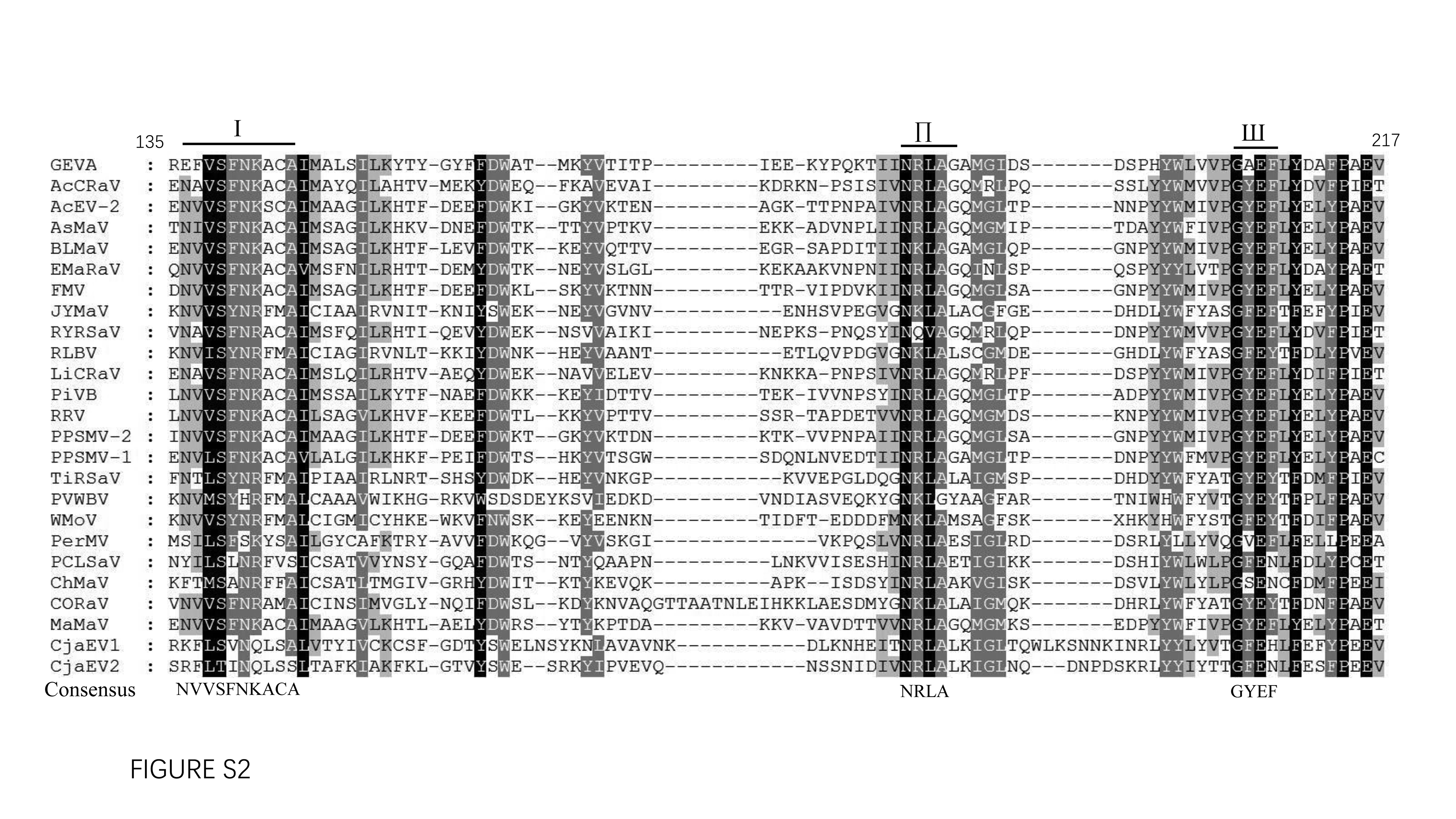

Supplement: Supplementary Figure 2 — Conserved motifs in P3 of grapevine emaravirus (GEVA) and other emaraviruses. [file Image_2.TIF]

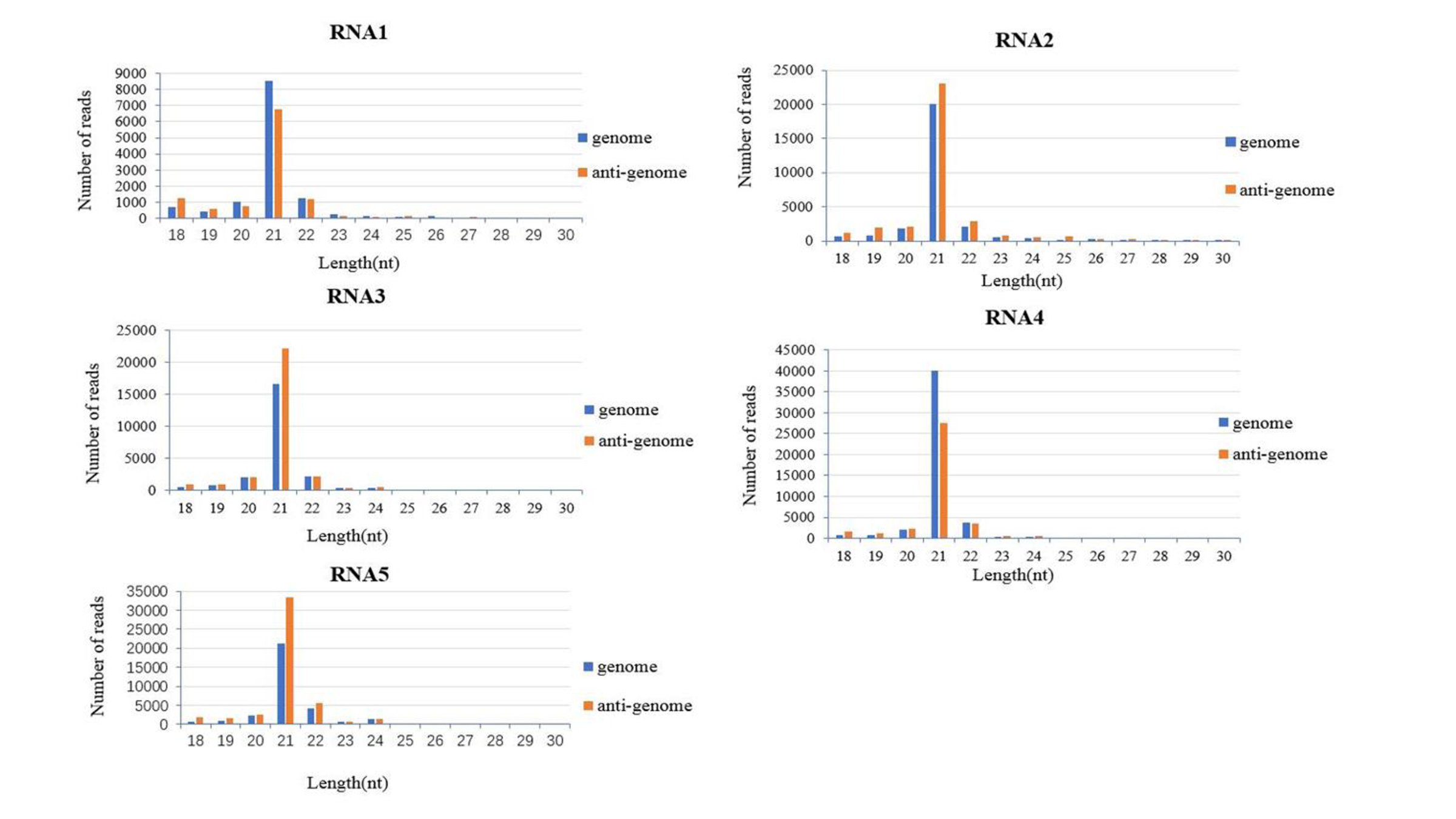

Supplement: Supplementary Figure 3 — Size distribution of 18- to 30-nt sRNAs derived from grapevine emaravirus A (GEVA) positive- and anti-genomic RNA strands. [file Image_3.TIF]

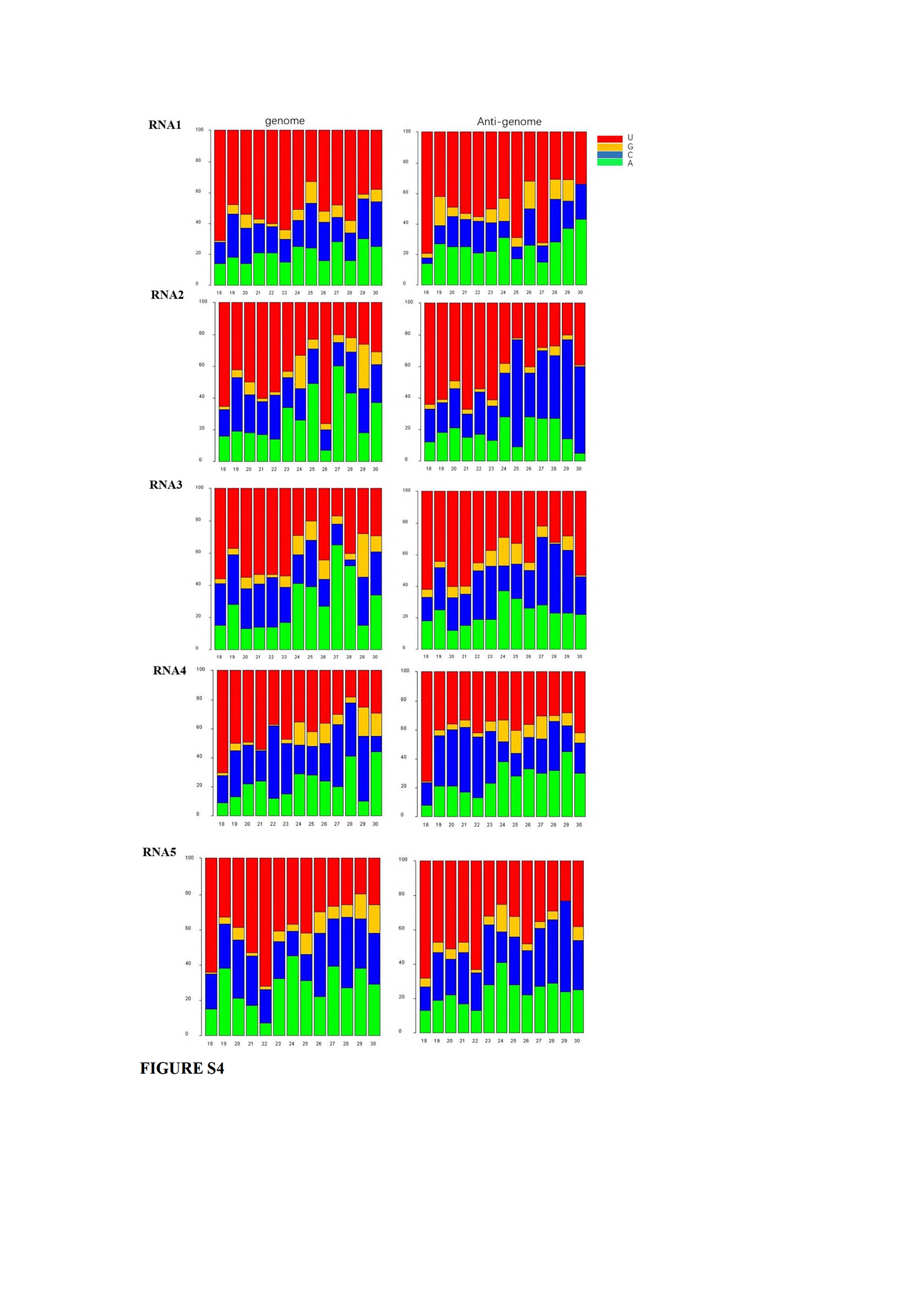

Supplement: Supplementary Figure 4 — Relative frequency of 5′-terminal nucleotides of 18–30 nt grapevine emaravirus A (GEVA) sRNAs. [file Image_4.TIF]
