# Supplementary material for: Identification and Characterization of a Novel Emaravirus From Grapevine Showing Chlorotic Mottling Symptoms
Source: Front Microbiol. 2021 Jun 7;12:694601. doi: 10.3389/fmicb.2021.694601 (PMC8215277; doi:10.3389/fmicb.2021.694601)
Supplement: Supplementary file 5 [file Table_1.DOCX]

**TABLE S1** Primers designed for PCR amplification of the full-length cDNAs of GEVA

| **RNA** | **Primer name** | **Primer sequence (5′→3′)** | **Position** |
| --- | --- | --- | --- |
| **RNA1** | R1-1F | GAATATGATTAGAATATATCATC | 122–144 |
|  | R1-1R | GATATCTTAGTGTATACCTATTG | 2123–2101 |
|  | R1-2F | AAACATTGTTGTATTTGAGTATCGAC | 1954–1979 |
|  | R1-2R | GGTTCATCTTCTACTTATTTTCAAG | 3551–3527 |
|  | R1-3F | TGATGACAACATGTTTAGATTCC | 3358–3380 |
|  | R1-3R | GACTCAATCTTTAACACAGTTGC | 5835–5813 |
|  | R1-4F | AGATAGGAATGCAATTTGCTTG | 5686–5707 |
|  | R1-4R | AATTCTTAACAATATTTGGCAAGAC | 6956–6932 |
|  | R1-5′ outer | TACAACAGGCTACTATGATAATCTC | 325–301 |
|  | R1-5′ inner | CATGATTGCATCATCTAGTGTTGC | 250–227 |
|  | R1-3′ outer | GATGCTCATTTGGTGGACAGTAC | 6797–6817 |
|  | R1-3′ inner | CCTTTCACATATTGAGATGACATCC | 6823–6847 |
| **RNA2** | R2-1F | GTAACCATTACATATGATCTTTG | 423–445 |
|  | R2-1R | CTAGACTGGTTAATGTCATGTCAAG | 1963–1939 |
|  | R2-5′ outer | CCAGACACACTATGTGGAAGTG | 560-539 |
|  | R2-5′ inner | GGAAGTGTAAATTGCAAAATAGGC | 545–522 |
|  | R2-3′ outer | GCTATTGTGAGACCTGACATATGG | 1801–1824 |
|  | R2-3′ inner | GACATATGGCTACAACTGGCATTG | 1816–1839 |
| **RNA3** | R3-1F | GATTTTCCCATTTTAATTCTC | 447–467 |
|  | R3-1R | GGCTTCAGGTTCAAACTTGAAAC | 1478–1456 |
|  | R3-2R (matching 5H) | CTGGAAAAGCTAAGAGAAACCAGG | 657–634 |
|  | R3-5′ outer | GATGTTTAGTTGCTCATTTATGTGTGTC | 571–544 |
|  | R3-5′ inner | CAAAAAAAACAAAAAAATACACAACCCG | 139–112 |
|  | R3-3′ outer | GTTGAGCTCCTTAGGTACATCAG | 1340–1362 |
|  | R3-3′ inner | GGTACATCAGCTGTCTTTGGATAG | 1353–1376 |
| **RNA4** | R4-1F | GTCAAACTAACATAGATATTAAAC | 356–379 |
|  | R4-1R | ACACTAAGACCTTCAACCATC | 1491–1471 |
|  | R4-2R (matching 5H) | GGACCATCAGACTAATCTTCATG | 487–465 |
|  | R4-5′ outer | GAAGAACAAAGGGAAAAGAGAG | 196–175 |
|  | R4-5′ inner | GAAAGTGAAGGTATGGCATGC | 134–114 |
|  | R4-3′ outer | GGTTTGTCTCTGACCCTTTATAAC | 1292–1315 |
|  | R4-3′ inner | GCCAAGCTTGGCAGGAATTTGTAG | 1367–1390 |
| **RNA5** | R5-1F | CTCATTGAAGAGAGGTTGTTTTG | 650–672 |
|  | R5-1R | GAACTGGAGACAAGCTTCTTTAG | 1130–1152 |
|  | R5-2R (matching 5H) | GTCAAGGTTGCTTGTCGCCGAATG | 789–766 |
|  | R5-5′ outer | CAACTAAACCGAACTAAAGATAATTGA | 144–118 |
|  | R5-5′ inner | CCGAACTAAAGATAATTGATATATAGGA | 136–109 |
|  | R5-3′ outer | CATCAACATGCTCTGTGACATC | 1011–1032 |
|  | R5-3′ inner | GACATCAATGAGGTCACTTAGAG | 1027–1049 |
|  | 5H | CTCAGCAGTAGTGTTCTCC |  |
|  | 3C | CTCAGCAGTAGTGAACTCC |  |
