# Supplementary material for: Identification and Characterization of a Novel Emaravirus From Grapevine Showing Chlorotic Mottling Symptoms
Source: Front Microbiol. 2021 Jun 7;12:694601. doi: 10.3389/fmicb.2021.694601 (PMC8215277; doi:10.3389/fmicb.2021.694601)
Supplement: Supplementary file 6 [file Table_2.DOCX]

TABLE S2 Contigs derived from the “Shennong Jinhuanghou” sample by small-RNA sequencing (sRNA-seq) and RNA-sequencing (RNA-seq) and matched proteins of emaraviruses as analyzed against the databases available in GenBank

| **Contig ID** | **Size(nt)** | **Virus** | **Protein/RNA** | **Position(aa)** | **Sequence identity (%)** |
| --- | --- | --- | --- | --- | --- |
| **sRNA-seq** |  |  |  |  |  |
| CONTIG222 | 64 | PPSMV-2( QBA83607) | RdRP | 1203–1222 | 85 |
| CONTIG151 | 59 | PPSMV-1(ANQ90727) | RdRP | 624–642 | 89 |
| CONTIG4 | 79 | FMV(AEI98677) | GP | 204–226 | 82 |
| CONTIG207 | 97 | FMV(AEI98677) | GP | 401–430 | 63 |
| CONTIG238 | 88 | FMV(AEI98677) | GP | 462–488 | 62 |
| CONTIG89 | 64 | RRV(QID76040) | GP | 150–168 | 89 |
| CONTIG125 | 192 | PPSMV-1(CCP46990) | GP | 13–64 | 53 |
| CONTIG12 | 243 | BLMaV(AQX45475) | NP | 90–166 | 51 |
| CONTIG133 | 155 | BLMaV(AQX45475) | NP | 173–223 | 58 |
| CONTIG127 | 252 | PPSMV-1(ANQ90752) | MP | 85–166 | 51 |
| CONTIG62 | 211 | FMV(BAM13822) | MP | 300–358 | 38 |
| CONTIG97 | 391 | PPSMV-2(ANQ90763) | P6 | 63–195 | 24 |
| **RNA-seq** |  |  |  |  |  |
| CONTIG567 | 374 | RRV(AXI82304) | RdRP | 8–130 | 45 |
| CONTIG788 | 2069 | RRV(AXI82304) | RdRP | 131–820 | 48 |
| CONTIG179 | 1479 | RRV(AXI82304) | RdRP | 823–1306 | 53 |
| CONTIG1378 | 271 | RRV(AXI82304) | RdRP | 1306–1394 | 77 |
| CONTIG537 | 761 | RRV(AXI82304) | RdRP | 1394–1646 | 59 |
| CONTIG387 | 2057 | RRV(AXI82304) | RdRP | 1642–2294 | 38 |
| CONTIG87 | 2080 | PPSMV-2(CCV01187) | GP | 1–337 | 44 |
| CONTIG114 | 1176 | PPSMV-1(ALU34073) | NP | 1–308 | 39 |
| CONTIG461 | 1325 | PiVB(QAR18005) | MP | 1–366 | 36 |
| CONTIG99 | 437 | PPSMV-1(ANQ90719) | P6 | 13–117 | 35 |
