# Supplementary material for: Identification and Characterization of a Novel Emaravirus From Grapevine Showing Chlorotic Mottling Symptoms
Source: Front Microbiol. 2021 Jun 7;12:694601. doi: 10.3389/fmicb.2021.694601 (PMC8215277; doi:10.3389/fmicb.2021.694601)
Supplement: Supplementary file 7 [file Table_3.docx]

**TABLE S3 Contigs from sRNA-seq and RNA-seq**

| Contigs | sequences |
| --- | --- |
| sRNA-seq |  |
| CONTIG127 | GTAGCTGTTGCAATGTCCATCTGCCCTGAGATCAGCTGTTGGAAATTAGGGTCAAATGTAACAACCTTTAGGATTCTGCCATCCATATTCTCAGCAAGTTGTTCACCAGTTGTTGATCTAGTAACACTCTTCAGCCCAGATTTGTTCATTCTAGCATCAATCACAAACAATGAAGCATGACTAGTAAATGATTTGCTTGTTGGAGTCCAAAAACCCAGTATGGTTGCCAATCTGGTTTGTCTCTGACCCTTT |
| CONTIG4 | ACACAGAGATCCAAATTTTAGACAAAGAGAACTACAAACAGGATTTTGATGTCAATGGTTATTTATATACATTTGTAGT |
| CONTIG207 | TATGGTCAGTCAGCTCAAGAGATCTAATATCAGAAACACCAGGGTCATCACAATGTTGTAATATAAATTCTCTATCAGCTGTGTGTGTGTCAATTAC |
| CONTIG238 | CTTCTGTGAGAGAGAAGTTGTAGTTAAGCGGATTGAAGTAACTGGGTGTTTTGACTGTTCAAGTGGGTTTGAAGCAAAAGTAGTTATT |
| CONTIG62 | AATTGAAGAAGCAATTCGATGACATAGAATCTGCTAGGATAAAGTTGATGAGGGCTGCTAATGGAGAAGATATTAGTTCAATTGAAAAGGCCCAAAAAGAAATGAAAGATGTAACTGGGAAATATGGCATAACAGTTGACGATGAGGAGAATGAACAAGGCTACATCAAGATAGGACCATCAGACTAATCTTCATGTATCCAGCTGTTACT |
| CONTIG97 | ACCTCATTGATGTCACAGAGCATGTTGATGTTAATGGACACATGGTCTCTAGAGGGGCTGTGAAGATGTTTCTATGCTACCTGCAGATAACATTGGCTCTCTTGGGAAACAAAGATGTGAATATATCAATTATCGATCCAAGGTTTAGAATCTTTGAGGATGTACATGCTCCTGACTTAAGTGCTAATCTTTTGTCAGGGAAAATGGCTATTGACAAACTGATAAGAGATGTTGTCATCAATGGCTGTAATGTCAAGGTTGCTTGTCGCCGAATGCTTAGGTCCATCACAACTAGTTTAAGTGTTCCAGAAGATTCTGTTGCTGGCTCCATGAAAGTCAAGGTCTATTGCTACCTTTGTTTGAAGAATCAAAACAACCTCTCTTCAATGAG |
| CONTIG12 | CTGCAGCTAGCTATCTTTCTCAGAGCAAAGAACTTAAAGAAAACTTGAAGAGGAAGGAATCACTGAGGCTTGAAGTTAGCAATGAGAACTATTTAATTGTAGTTCCTGATTTGACAGATTCAAGTGTTCGAGAATTTGTATCCTTCAACAAAGCTTGTGCGATCATGGCTTTATCTATCCTGAAGTACACTTATGGATACTTCTTTGATTGGGCAACAATGAAGTATGTAACTATAACACCAA |
| CONTIG133 | CCTATGATGTAGCTTGACCAGAACCAGTGCAATAACCTCTGCAGGGAATGCATCATATAGGAATTCTGCACCTGGAACAACCAGCCAGTAATGTGGGCTGTCACTATCGATTCCCATGGCTCCAGCTAACCTGTTGATGATTGTCTTTTGAGGAT |
| CONTIG125 | CACCTATATACCTCTCACCGTTGCATAAAGCTATTGTGAGACCTGACATATGGCTACAACTGGCATTGTACAAATAGTCACTGATAGGGCTTATCTTGCAACCATCAAAACACATCAAGTACATATTGCTCTCAAAAACAATATGTGGGTTGCAGACACAACTTGTTCTTGACATGACATTAACCAGTCTAG |
| CONTIG222 | TGATGACAACATGTTTAGATTCCCTTGTAGCCAGTTGCTCCTAACTTTGAACCAGTTTCTCTGA |
| CONTIG151 | TTATATGGGATAGAATACAGTTTCAGAATTTCTTGTGGTGAACCATGTAAGCCTTTATT |
| CONTIG89 | ATTTAAAATGTTCTAAATGTGATCAAAAATATCTATTCAAACATGTAGACTGTCCGACTCCAGT |
| RNA-seq |  |
| CONTIG87 | GTAGTGAACTCCTCAAATCAATCTATTCTTGATCAAATCTTACAAACTGTCAAAATGAGAATCACATCTGCATGGAACGTTGGTGTTGGATTGTTTGTTATATTGCTATACATGGTTTATAACTTGCCTGTAGCTAAACTGGTTAATGTCATGTCAAGAACAAGTTGTGTCTGCAACCCACATATTGTTTTTGAGAGCAATATGTACTTGATGTGTTTTGATGGTTGCAAGATAAGCCCTATCAGTGACTATTTGTACAATGCCAGTTGTAGCCATATGTCAGGTCTCACAATAGCTTTATGCAACGGTGAGAGGTATATAGGTGTTAAACCTGAATTGAAGAAAGATGAAGGCTTTACTGTCAATATCTTTTTGTCATGTATCAAGAAACTATTACTAGTCTTGATATCAACCATTAGTGCTTGGTTCATGCAGAAACCATTCTTATTTATATCATCTTTTGTACATTCATTGATGATCTCAAGGTCAAATTTAAAATGTTCTAAATGTGATCAAAAATATCTATTCAAACATGTAGACTGTCCGACTCCAGTGGCAAGAGATAGGACTGATTTAAATCTATTATTTTACCTTTTACTATTCTTCCTTGTTTTTACTGTTAGTATTAAGGCAGACGATAATGTATATAATTATTATGCACATGGTAACCACACAGAGATCCAAATTTTAGACAAAGAGAACTACAAACAGGATTTTGATGTCAATGGTTATTTATATACATTTGTAGTAAAAAATTCACACCTTATTGCAAACACTGTTAACATGTCAGTCATAACAGCTCCACAGAAGCACCACGTTGATCGAATATCTTGGAGTTGTGATGGCAATTCAGGCTGTTCTAAGGACCATGTGAACAAGTATGGTCTAGAGCCATCTTTTTACGTCAAGAAAGTTAGAGATGGTTTCAGCTGTATATTAACACAAGCCACAATTTGTGGTAATTGTCATTCTGACCACAATCCAATTGGTACCAAGGTTATAGTTAGCACATATGAACCGTATATTGAAATAGATGTTACACATGGCAATAAGACTGAACATATCTTGATCACAGATTTTAACACTTATATTCATAAGCCTTATTATGTAAAACCAATTCAAGGTCTTTATGTAAATACAAAAGAATATCTTGTGGTTGGCAAGTCTGTTTATAATGGAGTCTTCTGCTACAACCAGCATATGGTTGTTTTGGACCTAATTATATAAAAGATGACAAACATTTTAAATTGATTAATCCTAAAGTAATTGACACACACACAGCTGATAGAGAATTTATATTACAACATTGTGATGACCCTGGTGTTTCTGATATTAGATCTCTTGAGCTGACTGACCATATCTACTTTAATTCTACGATCATTATTCCACATTCTTTTGGTCTTATATCAATTGGTATACCTACATCTGGGAAACTAATTGGTGACTTCTGTGAGAGAGAAGTTGTAGTTAAGCGGATTGAAGTAACTGGGTGTTTTGACTGTTCAAGTGGGTTTGAAGCAAAAGTAGTTTATAAGCCAGACACACTATGTGGAAGTGTAAATTGCAAAATAGGCAACCTCAAGTACAAGTATTATGTAAATGTTGAAAGTGATTCTATAACATTACATTCATACACAGATAAACCAGATACAAAGATCATATGTAATGGTTACATAGACGTTGTTCAACTGAGCAAGCCATTAGACTCAGATTATGTATTATTATCAAATCATATCCACTCTGAAGCAGGAGATATCAAGCATTGGAACCTATTTCCCTTATTTTTCACTGACATAAAGTATTGTTCTTTAATGTGTTTGATTGCTTTGGTAAGCCTTTATCTATTACTAAGATTCTTTCTGAGGGTCAAACATTTCACTAGGAAGTTGATTTATAACAGAGAGGTAATAAAAATGAAGAAGGATGATGAGTACACAAGCGAATACATAGTTGTTGGTACAGCTGATTAAGCCTCTTACTATGTATATACTAAATTGGTAAATAGAGTTTTGCGTTTTCCATTATTTTGATTCTCACTTTAGATTATTTACTTTTTTTCTTCAATTCGTTTGGTATATTT |
| CONTIG461 | CAGTAGTGAACTCCTTACATAAAAATTATTCTTGAAAGAAATTTAATCATAATCTGGTAAGAGATCATTACAGCAAGATGCGTTGCATAATCTATTTCCTAATTGCCATCATTGCCATTTCACTGACAATGAAGAAGGTGAAAGCTGATGACACTAAGACCTTCAACCATCAATATATCGGGGAGATAGGCTTTGATTGGTCTGAGATGACTGAATTAGTCTGGGATTCAATTACATCAATGTCTTTAAAGCTACAAATTCCTGCCAAGCTTGGCTTAAATAAACTACCAATTAGTGTCTATTCTTCAATGTACAAGCTTTATAAAGTTATAAAGGGTCAGAGACAAACCAGATTGGCAACCATACTGGGTTTTTGGACTCCAACAAGCAAATCATTTACTAGTCATGCTTCATTGTTTGTGATTGATGCTAGAATGAACAAATCTGGGCTGAAGAGTGTTACTAGATCAACAACTGGTGAACAACTTGCTGAGAATATGGATGGCAGAATCCTAAAGGTTGTTACATTTGACCCTAATTTCCAACAGCTGATCTCAGGGCAGATGGACATTGCAACAGCTACACAGGACATTGACAAGTTGGAGTTCTATTTGACATTACCAATGTCTGGAATGGAATCTACTAATTCAGTAGGTGGCTTTTTTGACATCACTTGGAAAACACTTCCTGATGTAACAGGTGTCTATGAATCTACCAGATGGGATGTGTTCACATTCCCAAGAAGAACACCACCAGAGATTGAAGCTCAAGTTGGTAGGACCACTTTTGAAAAGCTAAAGAAATACTTTCAAGGGCAACATCAGAGTCAGGTTGAAAAGTTTAATAGGTTTGAAGAAATCTCATCACAGATTGCTCTTGGTAGCGATCAAGGTTTGAATAGGTTTAAAGAACAAGTCAAAGAAGCACATGCTTCAGCAAGCAACATGATGACTGATGCTAGCAGAAGGATTGAAGCAATAAAATTGAAGAAGCAATTCGATGACATAGAATCTGCTAGGATAAAGTTGATGAGGGCTGCTAATGGAGAAGATATTAGTTCAATTGAAAAGGCCCAAAAAGAAATGAAAGATGTAATGGGAAATATGGCATAACAGTTGACGATGAGGAGAATGAACAAGGCTACATCAAGATAGGACCATCAGACTAATCTTCATGTATCCAGCTGTTACTACTACTAGTATTTGTCTTTATATCACTGGTGTGTTATGTAACTTTGCCTAGTATTAAGATGCCTATTTATGTTTAATATCTATGTTAGTTTGACTAATCTAAATGAATGAAATAATAAAAAAATCACAAAAAAA |
| CONTIG114 | CTTTTTTTGATTTTCCCATTTTAATTCTCTTATTATTTATATATACTTATATTCATATAACATTACTTCACCATTAACCAATATTGCTTTATATTATAATATATAGACACACATAAATGAGCAACTAAACATCATAAACTCACTTGCTTGATTCCTGTATCTTTGCAATCAAGAGCTTGAAACCTTCCACCACATCCTGGTTTCTCTTAGCTTTTCCAGACACGCCTATGTTCTCTGTAAGCTGCCTGTAATTCTCCATTATGTTATCAATCCCTATGTTCTCAAAGATAGAATAAACATTGCTGTTGGTTATTGCATGTCTTCTGTTCATTTTCATTGTTAATGAAGACACTATATCTTCATCTGTAAACAAGTCAGGGAGATTGATATCATGCCTATGATGTAGCTTGACCAGAACCAGTGCAATAACCTCTGCAGGGAATGCATCATATAGGAATTCTGCACCTGGAACAACCAGCCAGTAATGTGGGCTGTCACTATCGATTCCCATGGCTCCAGCTAACCTGTTGATGATTGTCTTTTGAGGATATTTTTCTTCAATTGGTGTTATAGTTACATACTTCATTGTTGCCCAATCAAAGAAGTATCCATAAGTGTACTTCAGGATAGATAAAGCCATGATCGCACAAGCTTTGTTGAAGGATACAAATTCTCGAACACTTGAATCTGTCAAATCAGGAACTACAATTAAATAGTTCTCATTGCTAACTTCAAGCCTCAGTGATTCCTTCCTCTTCAAGTTTTCTTTAAGTTCTTTGCTCTGAGAAAGATAGCTAGCTGCAGCATCAATGTTGCAGTATGTTTTGTAATCCATGATGTTAAATTTCGATTCGAGTTGTTGCTGGCCTCGAAATGCCTTTTGCACATCTGCAGTTTTGATGTTGAGCTCCTTAGGTACATCAGCTGTCTTTGGATAGCTTCCATTAACAACATTTTGTAGAGTGATGACTTTCAATTTACCACTAGTACCAAATCTAATTGTAGCATCACCAATTGGTTTCAAGTTTGAACCTGAAGCCTTTGATATCATACTGCTTCTATTAGGTTTTGGAGGCATGGTTAATCTTCTAATTAACGTAACCTTGAATTAAAGTTTACCAGTTTAAAAGATGTTAAATTTTCTGAAAGAATAATTATGATTTGGGAGTTCACTAC |
| CONTIG567 | GTCAGGGAAAACATTTTTTATCTTTTCATCACAGGCATCAATCTTATAACCAGCTCTCTGTAAACTTAACATTATCACTCTCATCAATTCATCATGTCTTAGGAATTCAAGCATTATTATCATCGACACTGCAATATCAATTTGATGCTCATTTGGTGGACAGTACAATATCCTTTCACATATTGAGATGACATCCTTAGTGAAATTAGGATCTTGGATACAATCGTTGTATGCATGCTCAACATCTTTCCTCCTATGGCTTAGTGTGTACTTGCTTTTTGTCTTGCCAAATATTGTTAAGAATTTCTTTAGTATATCATCGTCTATGGCATCACCAGATCTAATTTTCTCAACAGCTTCGTTCAATACCTTTG |
| CONTIG788 | AATTTCTTGTGGTGAACCATGTAAGCCTTTATTACCGAGGTAAAATAATATAAACGCTTCGTGTATTATCTCTTTGGGATTATTTACAGTTAGATTGCTAATTGGTAATTTAAGATTTATATGTGGATTAAAACCTGTTGATATCAATTTACCATTCTCGTCAAAGTCACCTTCATTCCTATTTACATTTATAGTTTTGAGTTGATCGTTGGCTTTCATGAGACCATTTAACATGCAATTCAATATATATAGTTGCCCAATAGATTTTGGTCTAGCCTCTAGTTTGTCTTCAATCAAATCATCGATATTTGAATAATCAGAGTATATAGCCATTATTATGTTCTTGTAAGTGTCTGTAATACTTAGTGATGATATAGTTACAAACTGAGATAATAACCATGATATGGTGTGCACTCTATAAGGTATTTTACTCTTGAATTGTATATAGTGAGAGATCAACAAAGAGTATTTAACAAATGAGTGATTGAGTAGTTTCAGTCTAGTGACATCTAGACTTATAACTTTGCTAATCATTATTGTGTATTTATTAGATTTGCAGTGGGTATGGTACAAACCTAACAGCTTATTTGCTTCGATACTATCCACATCCTCTTTATCTAACACAGATATAATGAAATACCTGAGTGGACTTCCCTTCAAAGAATCTGCATTTGGCAACATTATCATTATATTACCTGGATCTTGACTTTGTATTAATCTATATTTATGGGAGTTGATAGTGTTGAGTGCAATCAATGCTTTATATTGATTATGCATGGAAAATAGATGTTCTAAATATTTTGTGCATGATTGCTCAGGTATGCCACATTTAACATTATTTACATTATCATTATCCAATGAAACTAAATCATTGGCATAAACACCACAGTGGTGTGGACTAGAAAATATTTTAGACAGATGTTTATCTAACAGTGCAATGTTTTCTAAGTTGTCAGATAATGATAGGTATTCCTTATTTTTTTCATTAGGATCATAGTGCTTGTTCACATAACTATTGATTGCATCTTTAGAATCCTTCATTAAATGATCACTAATAGAAAATGAATTGTTAGATAATATTGTTACATCACTACTCTGCCCAGATAGGAATGCAATTTGCTTGTATTTCTTATCATTGTCATTTATGGATTTTATGAACTCAGGTGTTAAAAATTGCTTGTAGTTTGGGATATCTATGTCAGAATCCTTGGTCACTAATATCTCAATTGCAACTGTGTTAAAGATTGAGTCAATTAGATTCACAACTGCGTTTGTATACCTTTCAGATGATTTACATGATATACCTTTGAATGCTTCTCTGTAAAATTTTATCCTATTACCACTATAATCCTCTAGCTTTATGGTTTTAGTTATAGGCAAATATATCGAAGGACGAAGTTTATCAGTTTTTTTGTATTTTAACATATTTTTTTTGTCAATCATGTCATATATGGATCGATTTTTTATCTGAGTCTGGGGGTATTTGTCTTTGTACACATATTCTTCATAATGATAAATAAAATTCTGGAGTGAGTCAGAGCATTCTTGGTATAAATCATTCCTTGAGTTAATCAAATCTTGGGTAACTTTTTCTTCATTTTCATATATTGAGAAATTTTCAACTTTGTCAATTAAATCTGTCCACCTACTGCCAAACAATCTCTTTATTTCACCATAATTTTCATTCCTTTCAAACATACCTCTATAACCATCAGGAATGAAGGCCACTTCATCTGTGTTGGATTGCAACCTTGGTGTGTAAATTGTATATTGTGGATACACCTCATATTTTGTCCTTATGTCTATACCCAGTTTCATTAGATAAATGATATCGTTGAATATGGAGTCAGTATCAATTATTTCACTTATCTTATAATCACCCTCTTCAACAAATCTGTCATAACAGAGATTAAATACTGCAACGTACACACCTTCTTGTTCAACAACATCTTTATATCTGTTATAATAATATTTCAAGTCTGTGTATTTGTTTCTAACCTTTATTTCAGCAATATACCTCTCTCCATCCTTTGAGAAGTATATATCTGGTGTCAGTATGCTATTGACGT |
| CONTIG179 | TTTTTTTCATTTAAAGTTATACAATGTTTTTTATTAGAAAAAGTGAGTAGTGATATTATAAATCTTCCAACATTTTGTTTTGATATCATGTCACGATAAGATTTGTCTGATTGACTATCTTGAATGAACAGGAAATCATAAGTTGAATCATCAGAGTGAACCATCGAAGTCATTACACATTCTACACCAGCTTCTTTACTAAATACATCAAGCATGAGCTCTGTGTACAGTGTGCTACAATAGTGAACATATGATGACAACATGTTTAGATTCCCTTGTAGCCAGTTGCTCCTAACTTTGAACCAGTTTCTCTGATAATTTGTTGTCATTTCTTCATATATGCCATGTTTACCTTCACATGCAAGTCTTATTAGATCATTATATGCATTATCTGTCAACAATATTTTTTTCCCATAATACCTTGAAAATAAGTAGAAGATGAACCATTTTTCGGATGAATACATATATGGATTACAAGCAATTGTCACAAAGAATTTTAGGAATACATCCCTAGCTGACCATTTGGATGCATCAGATGAAACTGATAGTAAATCAGATTTGGAGCCAGATTTTTTTGAAAATTTCATCTTTTTGAGAACAGCAATCCTCTGATCTAACACATTTTCCTCTGCTTTTGGTCACCAGATATAGTTATTGCTTCATTTGGAACAAATTTGTTTATAGCTTTATATATTTTCTCAGGAACATACAATGATAATCTAGTTTGAGCATTGCCAGTATAAATTTCTCTGTCTTCATATGTCCTTTGATCTTTATAGAATATCCTTATATTGAGATCACTTTCATGTATAACTTCATTATACATGTTCTTCAATACATTGACTTTACTCTCTTCTGTGATTCTATAAAATTCGTCAAAGACCTTTGCATTAGATTGCTTAACATATTCCCCATTAACCAGTTTAGAGTAATGTAGTCTGTTCAACCTGATAAATTCATTTCCATTTTTGAATTCATAATATACTGATGGTAAAAGCGTTATGCCTTTCTCCTTTGACAGTGGTTCAAGTAGAGATGCTAATGATGATTTTGAGCTTCTTAATCTATCTGTGTTTATACGATTTATTTCAGCATTTAGTTCTTTTGTAAATTGCTTTGCATCAACTATTTTGCTAGTATCTATAAATGTTTCTAAGCCATGAATATCAATCTTACCATCCAACTTTACAGGTTTGATTTCTTCAGATGGTATCCTATTTGAAACCATAGATTTTGTAGATGAAAATTGTTTTATTGACATAACATTAGTGTCCATAGAGAGTTCATCAACCATGCTTTTCCTGATCTGGTCCATTTTGCTTAGAAGTGTACTATATGAGTATATAGCTGTTTTTTTCATAACATCATAAGAAAATGACATGTTGTCCCTATTCCCATCTTCTTGTATAACTGTTTTGTACTTGTTTATGACTTCATCATACTCTTTTTCAAATTTATATGGGATAGAATACAGTTTCA |
| CONTIG1378 | AAAAAAAGACTTATATTAGTCGGTTTTATAAAGAGTTCTTATCAACAACGATTGTTAGCAATGAACTTTTCTTCTTTTATATGGCTGATCTACTCCCTATTGCCTCAGACACAACATACAAGTCACCACATGATGACCTAGCATCATATAGTGGATTCATTAACAATGCATTTTCACATGCATGTCCTTACAACATAATCAAGACAGCGATAACTTTAATAAATCACCTTATATTAAGCACATACAATCTCCAATACACAAGCAAAAAAAA |
| CONTIG537 | AAAAAAAATCCAAGACATATGATTGATACTAGTGACCTGCCTATTCAGATCTATCCAAGATATAAACTTAAAATGGATTTCGCTGGAACAATACCATACTATTCATCAGATGCAATTAACATCCTAGAGGACATGCTCTTAACGCTAGACAAATATAAAGATATTAAACACAATTTGATTGAAGATGTCATAAATAAAGATAGTGTCCAGTCATATCTAAACAAAATAGCTGGACAAGAAAAGAAAATAAATTATTTAAAATCATGTATGTTGTGTATGGATTACACTCAATATGATAGGGATGATGAAGACCCATACAACATTATAGATTATGACCTCAGCCAAAAGAGCATTATTAACTTGGTAAGCTTAAATAAAGGTCAAAGGTTAAAAAAAACATACACATACAATGAATATCTTAAGAATGAAAAAGATATCAGGCTCAAAACTTCAATAAATCCACTATGGTGTGTAAGCAAACCTAAAGATTTAAGTGATGTCAAGGTGCAAATACTGTCAAACTACTCAAATCCAATATTCAAAGATTCATTAATATACTCAACACCAGCAATAGACTATGGTCGTAGGGTCATAAGTTCTAATAGGAACGTTTACAAACTAACTAATACGGTGGAAGATAAAGGTAAAGCAAAGTCCATAACTGATGTTTATGGTATACTGGCTGATAAAATCAACAATTTAGAATTAGATGCTGACAGTATAATAAGATATCTTAGTGTATACCTATTGAGTGACAAAAA |
| CONTIG387 | AATATAGGTGATATAAATAAATCAGTATTGTTTAATTACATTGTGGCAAGTAATGATTGTTATGTCTATTTAGAATATGATTAGAATATATCATCATCATCCTGATTGATTGAATATGTGTCAGTATAGAATTTCAATATTGATATAATCATACAGATGATTTTCTGTTTTGGAGTTGCAACACTAGATGATGCAATCATGTACAGAAGATTTTTATAAGGTGATTTTTCATTCCTGTTATAAACATTATAGAGATTATCATAGTAGCCTGTTGTAAATTTTATCTTATGAAGATTTGATAACAATTTTGTTGGTTCTTCAATTTTGTTCAGATTAACCTTAGCCATTAGACATTCAACCTCTAAACAGTCAGTCAATTTACATAATATATCATCTTTAAACCCATTCTTCTTGTAAAAGTTCCCTATATTATATAATGAGACATATTTCATTACATCTATATTACTACAGAAGTCAACAAATACTTTTGTATGATTCATCAAATCTGAATAGTTTTTAGTTCCCATATAAGGATAAATGTCATCAAAGTACTCTTTATGGTTGATTAGATAATTTGAAGAACATAAATGATATGTGATATTATCTGGTGACAGTTCTATATTCTCTAGTAATGTTTCAATGTAAAAATCTGGGTCAATATAAATGCGGTTTTTTATGTCAAAAGTTATATCACCAGTTTGGGTTTTGAATTTTAATGATGTGAAAATATTATTTTCTTGTTCATAACAAACTGTTTCACAACCTAAAATATTTTGCACTGATATGTTGCTTATGTTGTTTCTTACAACATCATTCCTATTACAAAGTCTACCATATTGGTTGATATAACAATTTAAACCTTTGTTTATATTTGGCTTTGGGTGTATTATTGCCCTCCTAAATTGTTCATGGTAATCAGATGCTACTTTCCTCAATATTTGATCTAGAGTGTTGTTGTAATCTGTGTTTAAGAACTTATAGTAATTCATGACAAAATTAACTGCATTTCCGATTGATATAGTCTTGACAGACAATATATTCCCTCTACACATGTAGAGTGCTAAACTTGCATCGTCATCATCCTCAACATCTCCATGTCTCACAGGCCACATATTATAAACTCGATTATCTGTTGAAAGAGTTCTCATAAATGTTCTATAATCTATCTCACCAGTCATGTGGAGGTAACTTGTACTATTAATATCTGTTGTCAGTTTAAGATCTTTTATTAAATCGTTATAAGTATAATGACCAATAATGAATTGATCTCTCTGCTGCATGAGATCACTGTAAATCTCTTTATCATTTACAATTAATTTGGTAAGGAATGCTAGAGGTTCTTTAACACTTTTTTCTTTTCTAATGTCATGCTGCATGAGGTTAGATGTGCTAACTCTTATTTTATAACCAAGTTTAAATTTTGTATAGACATCTGGATCAGCATAGAACCCATATCTGGATAATAAATATTGATCGTATCTATCCGTTTGTGTTGTGGAAAGGTACACTTTACTAGATATCTGATCTTTCTTCATAAGTGAATCTATTGTCATGATTAGAGATGATGGTGATGGGTATGATGGTATATCATAATCTTTCTCATCAATGGCCTTTTGAGTGTCATTAAAGTATCTCACCAGCAACGACATCATTTTAATTTTATTTTTGTAGGCTTCAAAACCCCACTCATCTAGATTGTCAATTGGTTTTATATGTATGGCTGGTAGTTTATCCAAGTACTTGAAATTATAATAATTTATATAATCATCATCTATATCTTCTGGATCAGAGTAAATTTTTAAATCACCAATCCTATTTAGAACATATGTACAGATTGATACGAATTTTTGCACTTTTGAGTCAGCAAACTCCAATTCACAAAAATCATTAAGCAGCAGTTCGTCAAACATTGTTGTATTTGAGTATCGACCAAATTCTTCAGAGAACACACTTCTTGGAGCAACAACTCTAATAGCTTCCATCTTCGACATGACTAATACCTTCTGTTTCGAGTAATAAACCTGGACAGATAAAGATAATTTTTTGTCACTCAATAG |
| CONTIG99 | GTAGTGAACTCCCTATAACACATAGATTTGCAATCAAAGTTTAAGAAAATCACACTTAGGTAACTTCAATTGTTAAGCGTTTGATCATGGCATTCCAGATTCAAAAGCTCATGCTAGTCAATTGTACACTTGGCAATGTAAGTGACTACTATATTGAACTGGAGACAAGCTTCTTTAGTTCATTGGCTGAGAATATACCATCATCACTCTATGATGATGTCCATCTGCTCAGAGATGAATATGGTAATGGTGATTTATCTCTAAGTGACCTCATTGATGTCACAGAGCATGTTGATGTTAATGGACACATGGTCTCTAGAGGGGCTGTGAAGATGTTTCTATGCTACCTGCAGATAACATTGGCTCTCTTGGGAAACAAAGATGTGAATATATCAATTATCGATCCAAGGTTTAGAATCTTTGAGGATGTACATGCT |
