# Supplementary material for: Identification and Characterization of a Novel Emaravirus From Grapevine Showing Chlorotic Mottling Symptoms
Source: Front Microbiol. 2021 Jun 7;12:694601. doi: 10.3389/fmicb.2021.694601 (PMC8215277; doi:10.3389/fmicb.2021.694601)
Supplement: Supplementary file 8 [file Table_4.docx]

**TABLE S4** Identity of nucleotide and amino-acid sequences (%) between GEVA and other emaraviruses

| RNA | Virus | Virus ID | Genome |  |  | 5′ UTR | |  | ORF | | |  | 3′ UTR | |
| --- | --- | --- | --- | --- | --- | --- | --- | --- | --- | --- | --- | --- | --- | --- |
|  |  |  | Length | %^a^ |  | length | %^a^ |  | length | %^a^ | %^b^ |  | length | %^a^ |
| RNA1  (RdRp) | **GEVA** |  | 7090 | – |  | 129 | – |  | 6903 | – | – |  | 58 | – |
|  | AcCRaV | NC_038769 | 7061 | 56.4 |  | 111 | 51.4 |  | 6912 | 56.4 | 48.4 |  | 38 | 59.8 |
|  | AcEV-2 | MK602171 | 7079 | 57.8 |  | 142 | 49.0 |  | 6894 | 58.1 | 48.7 |  | 43 | 58.8 |
|  | AsMaV | LR742461 | 7106 | 56.8 |  | 149 | 47.6 |  | 6912 | 57.3 | 49.4 |  | 45 | 61.6 |
|  | BLMaV | KY056657 | 7050 | 57.8 |  | 93 | 51.4 |  | 6909 | 58.1 | 48.4 |  | 48 | 60.2 |
|  | EMARaV | LR536375 | 7040 | 56.8 |  | 114 | 45.8 |  | 6882 | 57.7 | 49.1 |  | 44 | 58.9 |
|  | FMV | MH475439 | 7039 | 58.2 |  | 106 | 56.6 |  | 6894 | 58.4 | 48.9 |  | 39 | 62.4 |
|  | JYMaV | MK305894 | 7143 | 48.5 |  | 112 | 52.1 |  | 6930 | 49.7 | 32.2 |  | 101 | 20.6 |
|  | LiCRaV | MT112174 | 7047 | 56.6 |  | 96 | 42.5 |  | 6897 | 57.1 | 47.7 |  | 54 | 58.9 |
|  | PPSMV-1 | MH374920 | 7023 | 58.1 |  | 89 | 35.5 |  | 6885 | 58.3 | 49.8 |  | 49 | 58.8 |
|  | PPSMV-2 | MH374925 | 70083 | 58.6 |  | 78 | 37.1 |  | 6885 | 59.0 | 49.4 |  | 44 | 60.2 |
|  | PiVB | MH727572 | 7027 | 59.2 |  | 75 | 41.0 |  | 6900 | 59.4 | 49.6 |  | 52 | 57.9 |
|  | RLBV | NC_029567 | 7062 | 49.7 |  | 126 | 52.8 |  | 6888 | 50.2 | 33.3 |  | 48 | 59.2 |
|  | RYRSaV | NC_038852 | 7049 | 55.6 |  | 109 | 40.6 |  | 6900 | 56.6 | 48.1 |  | 40 | 60.6 |
|  | RRV | MN106935 | 6980 | 56.4 |  | 67 | 35.0 |  | 6894 | 58.5 | 49.1 |  | 19 | 42.6 |
|  | TiRSaV | MH223635 | 7217 | 47.7 |  | 138 | 52.8 |  | 7023 | 47.2 | 34.3 |  | 56 | 58.9 |
|  | PVWBV | MF766024 | 7015 | 48.7 |  | 106 | 38.0 |  | 6849 | 47.9 | 32.2 |  | 60 | 58.9 |
|  | WMoV | KT988869 | 6972 | 47.7 |  | 85 | 40.9 |  | 6819 | 47. 3 | 31.4 |  | 68 | 59.6 |
|  | PerMV | LC496090 | 7291 | 49.6 |  | 204 | 33.5 |  | 7008 | 49.4 | 29.0 |  | 79 | 50.0 |
|  | PCLSaV | MK602177 | 7100 | 52.3 |  | 86 | 40.7 |  | 6945 | 51.6 | 32.1 |  | 69 | 34.5 |
|  | chMaV | LC576445 | 7093 | 36.4 |  | 120 | 58.1 |  | 6909 | 48.2 | 30.7 |  | 64 | 58.6 |
|  | CORaV | LR828198 | 7074 | 53.3 |  | 97 | 66.0 |  | 6954 | 49.6 | 33.3 |  | 58 | 66.7 |
|  | MaMaV | MT879790 | 7090 | 60.7 |  | 108 | 48.0 |  | 6918 | 60.6 | 49.5 |  | 48 | 64.6 |
|  | CjaEV1 | MN385573 | 7109 | 47.5 |  | 78 | 58.1 |  | 6963 | 48.3 | 27.8 |  | 68 | 50.0 |
|  | CjaEV2 | MN385577 | 7120 | 47.4 |  | 76 | 45.8 |  | 6975 | 49.4 | 27.9 |  | 154 | 53.2 |
| RNA2  (GP) | **GEVA** |  | 2097 | – |  | 125 | – |  | 1917 | – | – |  | 55 | – |
|  | AcCRaV | NC_038770 | 2267 | 33.9 |  | 251 | 42.0 |  | 1962 | 55.0 | 39.1 |  | 54 | 53.8 |
|  | AcEV-2 | MK602172 | 2252 | 37.6 |  | 266 | 34.9 |  | 1950 | 54.2 | 39.4 |  | 36 | 69.4 |
|  | AsMaV | LR742462 | 2288 | 36.2 |  | 305 | 22.4 |  | 1926 | 56.0 | 39.9 |  | 57 | 57.4 |
|  | BLMaV | KY056658 | 2271 | 36.6 |  | 265 | 34.3 |  | 1959 | 53.8 | 39.7 |  | 47 | 70.2 |
|  | EMARaV | LR536376 | 2335 | 32.4 |  | 336 | 16.8 |  | 1941 | 54.5 | 37.6 |  | 58 | 65.5 |
|  | FMV | MH580610 | 2253 | 39.4 |  | 275 | 34.2 |  | 1926 | 55.1 | 40.7 |  | 52 | 57.7 |
|  | JYMaV | MK305895 | 2233 | 36.4 |  | 190 | 62.3 |  | 1989 | 44.6 | 22.0 |  | 57 | 44.4 |
|  | LiCRaV | MT112175 | 2268 | 34.6 |  | 274 | 34.0 |  | 1953 | 56.5 | 38.3 |  | 41 | 46.3 |
|  | PPSMV-1 | MH374921 | 2223 | 37.6 |  | 235 | 46.7 |  | 1947 | 54.7 | 40.4 |  | 41 | 46.2 |
|  | PPSMV-2 | MH374921 | 2230 | 39.5 |  | 233 | 44.7 |  | 1950 | 56.1 | 43.1 |  | 41 | 46.2 |
|  | PiVB | MH727573 | 2245 | 40.0 |  | 266 | 32.9 |  | 1923 | 55.6 | 39.3 |  | 56 | 54.7 |
|  | RLBV | NC_029558 | 2135 | 39.7 |  | 133 | 78.2 |  | 1953 | 46.1 | 21.9 |  | 49 | 49.0 |
|  | RYRSaV | NC_038856 | 2220 | 37.0 |  | 244 | 45.4 |  | 1929 | 56.7 | 40.2 |  | 50 | 50.0 |
|  | RRV | MN120582 | 2189 | 39.1 |  | 236 | 38.9 |  | 1938 | 55.3 | 39.9 |  | 15 | 66.7 |
|  | TiRSaV | MH223636 | 2398 | 30.8 |  | 187 | 64.1 |  | 2154 | 35.2 | 24.8 |  | 58 | 48.1 |
|  | PVWBV | MF766029 | 2098 | 38.9 |  | 129 | 79.5 |  | 1920 | 43.5 | 21.8 |  | 49 | 42.6 |
|  | WMoV | KT988870 | 2211 | 33.3 |  | 128 | 77.5 |  | 2004 | 47.2 | 23.2 |  | 79 | 41.8 |
|  | PerMV | LC496091 | 2092 | 48.4 |  | 118 | 51.3 |  | 1920 | 47.9 | 19.9 |  | 54 | 60.4 |
|  | PCLSaV | MK602178 | 2045 | 48.3 |  | 136 | 39.2 |  | 1839 | 48.2 | 19.0 |  | 70 | 56.4 |
|  | chMaV | LC576446 | 2054 | 39.5 |  | 82 | 59.3 |  | 1902 | 39.3 | 17.8 |  | 70 | 44.8 |
|  | CORaV | LR828199 | 2310 | 41.9 |  | 283 | 49.6 |  | 2310 | 45.0 | 21.9 |  | 71 | 64.8 |
|  | MaMaV | MT879791 | 2289 | 55.8 |  | 293 | 48.3 |  | 2289 | 55.9 | 42.6 |  | 55 | 60.0 |
|  | CjaEV1 | MN385574 | 2054 | 43.5 |  | 29 | 64.3 |  | 2054 | 39.5 | 19.8 |  | 69 | 52.7 |
|  | CjaEV2 | MN385578 | 2089 | 43.7 |  | 28 | 64.3 |  | 2089 | 39.2 | 19.9 |  | 78 | 47.3 |
| RNA3  (CP) | **GEVA** |  | 1615 | – |  | 577 | – |  | 939 | – | – |  | 99 | – |
|  | AcCRaV | NC_038772 | 1678 | 41.8 |  | 645 | 36.3 |  | 933 | 45.0 | 35.2 |  | 100 | 46.3 |
|  | AcEV-2 | MK602173 |  | 43.2 |  | 335 | 36.0 |  | 951 | 49.0 | 41.5 |  | 101 | 49.5 |
|  | AsMaV | LR742463 | 1587 | 46.6 |  | 530 | 46.2 |  | 957 | 48.4 | 39.4 |  | 100 | 52.1 |
|  | BLMaV | KY056659 | 1510 | 46.1 |  | 462 | 54.3 |  | 951 | 47.5 | 40.7 |  | 97 | 42.4 |
|  | EMARaV | LR536377 | 1560 | 43.4 |  | 496 | 31.4 |  | 945 | 48.7 | 35.3 |  | 119 | 36.7 |
|  | FMV | MH569162 | 1491 | 44.4 |  | 444 | 41.8 |  | 948 | 49.4 | 43.5 |  | 99 | 43.6 |
|  | JYMaV | MK305896 | 1259 | 35.9 |  | 304 | 42.0 |  | 885 | 35.1 | 20.6 |  | 70 | 46.2 |
|  | LiCRaV | MT112176 | 1557 | 46.5 |  | 539 | 40.7 |  | 885 | 35.1 | 20.6 |  | 88 | 43.4 |
|  | PPSMV-1 | MH374922 | 1443 | 46.4 |  | 414 | 43.6 |  | 927 | 50.5 | 39.5 |  | 102 | 40.6 |
|  | PPSMV-2 | MH374927 | 1335 | 45.1 |  | 289 | 35.7 |  | 945 | 52.2 | 43.5 |  | 101 | 37.5 |
|  | PiVB | MH727574 | 1711 | 47.1 |  | 506 | 53.1 |  | 918 | 52.7 | 39.1 |  | 101 | 49.0 |
|  | RLBV | NC_029559 | 1365 | 35.9 |  | 433 | 41.7 |  | 879 | 35.6 | 19.4 |  | 53 | 52.1 |
|  | RYRSaV | NC_038854 | 1414 | 42.3 |  | 383 | 37.7 |  | 942 | 48.0 | 34.2 |  | 89 | 47.6 |
|  | RRV | MN133349 | 1493 | 46.2 |  | 471 | 61.1 |  | 951 | 47.1 | 39.0 |  | 71 | 31.8 |
|  | TiRSaV | MH223637 | 1101 | 29.4 |  | 117 | 65.2 |  | 861 | 38.6 | 21.6 |  | 128 | 43.6 |
|  | PVWBV | MF766034 | 1356 | 36.3 |  | 392 | 41.9 |  | 879 | 38.9 | 18.5 |  | 85 | 71.2 |
|  | WMoV | KT988871 | 1070 | 26.3 |  | 127 | 34.4 |  | 864 | 40.6 | 18.5 |  | 79 | 54.8 |
|  | PerMV | LC496092 | 1080 | 45.7 |  | 190 | 43.2 |  | 798 | 46.4 | 21.5 |  | 92 | 51.1 |
|  | PerMV | LC496093 | 1078 | 47.0 |  | 189 | 45.5 |  | 798 | 47.3 | 21.1 |  | 91 | 48.3 |
|  | PCLSaV | MK602179 | 1296 | 52.3 |  | 407 | 55.9 |  | 801 | 50.7 | 24.9 |  | 88 | 50.0 |
|  | chMaV | LC576447 | 1390 | 40.3 |  | 524 | 42.2 |  | 792 | 35.9 | 20.0 |  | 74 | 55.7 |
|  | CORaV | LR828200 | 1338 | 45.2 |  | 388 | 52.8 |  | 876 | 38.6 | 20.8 |  | 74 | 57.1 |
|  | MaMaV | MT879192 | 1525 | 57.5 |  | 519 | 64.0 |  | 903 | 50.5 | 39.7 |  | 103 | 67.1 |
|  | CjaEV1 | MN385575 | 1360 | 36.6 |  | 357 | 46.9 |  | 888 | 42.8 | 19.1 |  | 115 | 42.3 |
|  | CjaEV2 | MN385579 | 1316 | 38.1 |  | 300 | 39.1 |  | 900 | 43.6 | 20.1 |  | 116 | 43.3 |
| RNA4  (MP) | **GEVA** |  | 1640 | – |  | 472 | – |  | 1092 | – | – |  | 76 | – |
|  | AcCRaV | NC_038771 | 1664 | 40.1 |  | 426 | 36.1 |  | 1140 | 43.6 | 22.0 |  | 98 | 46.4 |
|  | AcEV-2 | MK602174 | 1514 | 48.9 |  | 346 | 37.9 |  | 1086 | 52.2 | 34.3 |  | 82 | 60.8 |
|  | AsMaV | LR742464 | 1557 | 46.3 |  | 385 | 38.4 |  | 1086 | 48.8 | 34.1 |  | 86 | 50.0 |
|  | BLMaV | KY056660 | 1540 | 48.1 |  | 332 | 46.5 |  | 1092 | 50.6 | 34.7 |  | 80 | 54.1 |
|  | EMARaV | LR536378 | 1347 | 30.7 |  | 503 | 40.0 |  | 699 | 36.2 | 11.2 |  | 145 | 51.3 |
|  | FMV | MH569168 | 1485 | 48.0 |  | 318 | 36.5 |  | 1086 | 50.2 | 34.6 |  | 81 | 58.3 |
|  | JYMaV | MK305897 | 1547 | 38.7 |  | 321 | 48.3 |  | 1125 | 38.4 | 14.3 |  | 101 | 40.8 |
|  | LiCRaV | MT112177 | 1657 | 42.4 |  | 416 | 43.7 |  | 1146 | 45.5 | 26.4 |  | 95 | 46.4 |
|  | PPSMV-1 | MH374923 | 1557 | 46.4 |  | 395 | 39.9 |  | 1086 | 50.1 | 34.6 |  | 76 | 49.3 |
|  | PPSMV-2 | MH374928 | 1487 | 47.2 |  | 319 | 39.9 |  | 1086 | 51.8 | 35.5 |  | 82 | 54.1 |
|  | PiVB | MH727575 | 1550 | 48.8 |  | 383 | 50.4 |  | 1086 | 52.0 | 32.4 |  | 81 | 55.6 |
|  | RLBV | NC_029560 | 1675 | 38.7 |  | 472 | 46.4 |  | 1122 | 38.0 | 16.0 |  | 81 | 47.1 |
|  | RYRSaV | NC_038853 | 1513 | 41.9 |  | 283 | 40.4 |  | 1131 | 43.0 | 23.1 |  | 99 | 50.7 |
|  | RRV | MN136330 | 1501 | 47.7 |  | 349 | 35.0 |  | 1086 | 50.4 | 33.8 |  | 66 | 38.6 |
|  | TiRSaV | MH223638 | 1342 | 36.8 |  | 262 | 42.4 |  | 999 | 38.1 | 15.4 |  | 81 | 40.3 |
|  | PVWBV | MF766039 | 1481 | 39.2 |  | 310 | 48.4 |  | 1101 | 39.1 | 15.7 |  | 70 | 52.2 |
|  | WMoV | KT988873 | 1681 | 37.8 |  | 474 | 39.4 |  | 1095 | 38.5 | 17.6 |  | 112 | 45.9 |
|  | PerMV | LC496094 | 1294 | 48.3 |  | 160 | 48.1 |  | 1032 | 46.6 | 18.0 |  | 102 | 56.6 |
|  | PCLSaV | MK602180 | 1543 | 49.6 |  | 464 | 48.2 |  | 966 | 47.7 | 15.0 |  | 133 | 30.3 |
|  | chMaV | LC576448 | 1303 | 34.3 |  | 524 | 42.2 |  | 927 | 36.4 | 10.8 |  | 70 | 46.9 |
|  | CORaV | LR828201 | 1411 | 39.2 |  | 388 | 52.8 |  | 1098 | 33.3 | 12.2 |  | 67 | 50.8 |
|  | MaMaV | MT879193 | 1533 | 59.7 |  | 519 | 64.0 |  | 1092 | 56.5 | 11.4 |  | 87 | 52.7 |
|  | CjaEV1 | MN385576 | 1349 | 41.3 |  | 357 | 46.9 |  | 1023 | 36.6 | 13.0 |  | 154 | 53.9 |
|  | CjaEV2 | MN385580 | 1154 | 41.5 |  | 300 | 39.1 |  | 1026 | 38.7 | 10.6 |  | 89 | 46.5 |

^a^ Nucleotide

^b^ Amino acid
