# Supplementary material for: Identification and Characterization of a Novel Emaravirus From Grapevine Showing Chlorotic Mottling Symptoms
Source: Front Microbiol. 2021 Jun 7;12:694601. doi: 10.3389/fmicb.2021.694601 (PMC8215277; doi:10.3389/fmicb.2021.694601)
Supplement: Supplementary file 9 [file Table_5.docx]

**TABLE S5** Summary of lllumina deep-sequencing data

|  |  | Reads | Percentage（%） |
| --- | --- | --- | --- |
| sRNAs | Clean reads | 14,348,642 |  |
| vsiRNA | Reads matching viral genome | 303,164 | 2.11 |
|  | Reads matching Genomic strands | 143,074 | 0.99 |
|  | Reads matching anti-genomic RNAs | 160,090 | 1.12 |
|  | RNA1 | 23,829 | 0.17 |
|  | RNA2 | 61,088 | 0.43 |
|  | RNA3 | 52,943 | 0.37 |
|  | RNA4 | 86,093 | 0.60 |
|  | RNA5 | 79,211 | 0.55 |
| RNA | Clean reads | 60,846,756 |  |
|  | Reads matching viral genome | 224,226 | 0.37 |
|  | RNA1 | 37,355 | 0.06 |
|  | RNA2 | 65,078 | 0.10 |
|  | RNA3 | 22,472 | 0.04 |
|  | RNA4 | 16,805 | 0.03 |
|  | RNA5 | 82,516 | 0.14 |
